# Synthesis of α,β‐ and β‐Unsaturated Acids and Hydroxy Acids by Tandem Oxidation, Epoxidation, and Hydrolysis/Hydrogenation of Bioethanol Derivatives

**DOI:** 10.1002/anie.202002049

**Published:** 2020-03-11

**Authors:** Daniel Santhanaraj, Maria P. Ruiz, Mallik R. Komarneni, Tu Pham, Gengnan Li, Daniel E. Resasco, Jimmy Faria

**Affiliations:** ^1^ Faculty of Science and Technology Catalytic Processes and Materials group MESA+ Institute for Nanotechnology University of Twente Enschede 7522 NB The Netherlands; ^2^ School of Chemical, Biological and Materials Engineering University of Oklahoma Norman OK 73019 USA; ^3^ Faculty of Science and Technology Sustainable Process Technology group University of Twente Enschede 7522 NB The Netherlands; ^4^ Present address: Department of Chemistry Loyola College Chennai 600-034 India

**Keywords:** bioethanol, chemical building blocks, polyhydroxyalkanoates, tandem chemistry, unsaturated acids

## Abstract

We report a reaction platform for the synthesis of three different high‐value specialty chemical building blocks starting from bio‐ethanol, which might have an important impact in the implementation of biorefineries. First, oxidative dehydrogenation of ethanol to acetaldehyde generates an aldehyde‐containing stream active for the production of C_4_ aldehydes via base‐catalyzed aldol‐condensation. Then, the resulting C_4_ adduct is selectively converted into crotonic acid via catalytic aerobic oxidation (62 % yield). Using a sequential epoxidation and hydrogenation of crotonic acid leads to 29 % yield of β‐hydroxy acid (3‐hydroxybutanoic acid). By controlling the pH of the reaction media, it is possible to hydrolyze the oxirane moiety leading to 21 % yield of α,β‐dihydroxy acid (2,3‐dihydroxybutanoic acid). Crotonic acid, 3‐hydroxybutanoic acid, and 2,3‐dihydroxybutanoic acid are archetypal specialty chemicals used in the synthesis of polyvinyl‐co‐unsaturated acids resins, pharmaceutics, and bio‐degradable/ ‐compatible polymers, respectively.

## Graphical abstract

**A flexible chemical platform** is described for the conversion of bioethanol into three distinct specialty chemical building blocks at high yields: crotonic acid (i, 62 %), 3‐hydroxy butanoic acid (ii, 29 %), and 2,3‐dihydroxyacid (iii, 21 %). The products can serve as basis for the production of numerous high‐value chemicals.

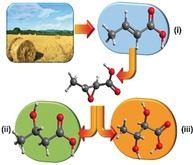

Polymers play a key role in the creation of a myriad of materials that have contributed to the development of our modern society. The current use of plastics, however, is not sustainable in the long term due to its dependence on non‐renewable fossil fuels and the environmental pollution caused by plastic waste.^1^ This dilemma has triggered intensive research in the development of environmentally friendly and sustainable bio‐based and biodegradable polymers.^2^ Polyhydroxyalkanoates (PHAs) are a class of biodegradable isotactic polymers synthesized by bacteria.^3^ Until now, these family of polymer building blocks have been synthesized using genetically modified micro‐organisms or enzymes.^4^ However, the production cost of PHAs is nearly four times larger than its petroleum‐based counterparts (circa PHAs^5^ and polypropylene^6^ prices are 5–6 and 1–2 $/kg, respectively). This is due to the high cost of raw materials, low conversion rates, the complex purification of the fermentation broths, and the large amounts of biomass waste generated (circa 5 kg of raw material per 1 kg of product), and low conversion rates.^7^

Catalytic conversion routes of biomass‐derived feedstocks to short β‐hydroxy acids (e.g. lactic acid) have shown higher productivities and atom‐efficiency at industrially relevant operating conditions,^8^ but their application has been limited to short chain (C_3_) molecules. Inspired by nature, we have developed a new catalytic cascade process that mimics the step‐wise coupling of C_2_ units that occur during the biosynthesis of PHA in bacteria. Accordingly, we have used base‐catalyzed aldol‐condensation followed by tandem oxidation, epoxidation and hydrogenation or hydration (Scheme 1). This catalytic cascade route has allowed us to generate medium‐chain α,β‐unsaturated acids (crotonic acid), α,β‐dihydroxy acids (2,3‐dihydroxybutanoic acid), and β‐hydroxy acids (3‐hydroxybutyric acid), which are emerging specialty chemicals and building blocks.

**Scheme 1:**
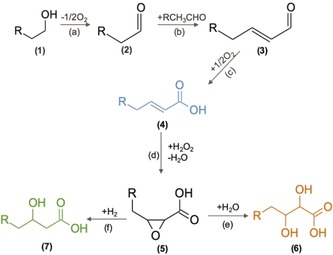
Integrated catalytic production of unsaturated, mono‐ and di‐hydroxy acids from bioethanol, via tandem oxidative dehydrogenation (a), aldol condensation (b), oxidation (c), epoxidation (d), and hydrolysis (e) or hydrogenation (f). If R is equal to H, then species involved are; (1) ethanol, (2) acetaldehyde, (3) crotonaldehyde, (4) crotonic acid, (5) 3‐methyloxirane‐2‐carboxylic acid, (6) 2,3‐dihydroxybutanoic acid, (7) 3‐hydroxybutanoic acid.

The process of converting ethanol into unsaturated and mono‐ and di‐hydroxy acids starts with the catalytic dehydrogenation of ethanol to acetaldehyde (Scheme 1 a). This step was accomplished in a flow reactor at 250 °C using SiO_2_‐supported 10 wt. % Cu and 5 wt. % Ni catalysts (see Table S1 in the Supporting Information). When the reaction was performed at conversions below 20 % in the presence of oxygen, the observed rates were 0.67±0.06 and 0.46±0.02 g_EtOH_ g_cat_^−1^ h^−1^ on the Cu and Ni catalysts, respectively. At these conditions the selectivity on both catalysts in the presence of oxygen was circa 100 %, which is in agreement with previous kinetic studies performed on Cu–SiO_2_, Cu–ZnO and Cu–Al_2_O_3_ catalysts that indicated that selectivities to acetaldehyde 80 % can be obtained below 20 % conversion.^9^, ^10^ The unreacted ethanol could either be recycled back for further reaction or kept in the product mixture for conducting the aldol condensation step (Figure S9).

To selectively convert the C_2_ aldehydes into the corresponding C_4_ coupling products (Scheme 1 b) we have developed a number of catalytic systems based on basic K/Na‐X and K/Na‐Y,^11^ and MgO‐K/NaX,^12^ zeolites as well as B‐doped MgO oxides.^13^ The Mg‐Al Hydrotalcite, Al‐Beta zeolite, which along with the Mg‐B catalyst previously employed have shown the highest C_4_ productivities among all the catalysts investigated, with productivities of 8.3, 23.4, and 24.2 mmol of C_4_ g_cat_^−1^ h^−1^, respectively, at 50–60 % conversion level and 180 °C (Table S2). Notably, on the 7.5 wt. % B‐MgO and Al‐Beta catalysts the yield of C4 unsaturated aldehydes reached 60–70 mol C % at levels of conversion (80–90 %).

The subsequent conversion of the C_4_ adduct to the corresponding β‐hydroxy acid is rather challenging as the dehydration towards the C_4_ α,β‐unsaturated acid is thermodynamically and kinetically more favorable under the reaction conditions required for the oxidation.^14^ To circumvent this limitation, we employed a tandem oxidation‐epoxidation sequence. First, to accomplish aerobic oxidation step we employed two distinct types of Ru catalysts supported on reducible metal‐oxides (5 wt. % RuCo_*x*_Ce_*y*_O_*z*_) and a microporous titanosilicate catalysts (ETS‐4) (Table 1). When the reaction was performed on the Co_*x*_Ce_*y*_O_*z*_ catalyst without Ru the conversion of crotonaldehyde was ≈66 % and the yield to crotonic acid was 54 % after 6 hours of reaction in decalin.

**Table 1:** Product selectivity and conversions obtained during aerobic oxidation reaction of crotonaldehyde to crotonic acid at 38 bar of synthetic air using 200 mg of catalyst in decalin.^[a]^

Catalyst	Reaction time [h]	Temperature [°C]	Solvent	Conversion [%]	Crotonic acid yield [%]	STY [mol g^−1^ h^−1^]
Co_*x*_Ce_*y*_O_*z*_	6	100	Decalin	66	54	0.03
RuCo_*x*_Ce_*y*_O_*z*_	6	100	Decalin	92	92	0.09
RuCo_*x*_Ce_*y*_O_*z*_	6	100	Water	78	9	0.15
RuCo_*x*_Ce_*y*_O_*z*_	6	100	GVL	67	54	1.53
RuCo_*x*_Ce_*y*_O_*z*_	6	80	Water	66	5	0.91
RuCo_*x*_Ce_*y*_O_*z*_	6	60	Water	48	2	0.96
ETS‐4	6	100	Decalin	70	58	6.07
ETS‐4^€^	0.75	100	Acetic acid	85	85	11.33

[a] RuCoCeO_2_ refers to 5 wt. % Ru supported on Co_*x*_Ce_*y*_O_*z*_ oxide. STY refers to the site time yield calculated as the moles of product per mol of catalyst per unit of time.

Addition of 5 wt. % Ru to the Co_*x*_Ce_*y*_O_*z*_ led to a significant improvement in the selectivity and activity of the catalyst that resulted in conversion values of circa 92 % and yield to crotonic acid of 100 %. The high activity and selectivity of this reaction could be attributed to the cooperative interaction between Ru and Ce sites on the CoO(OH) surface that facilitate the oxidation of the Co^2+^ to Co^3+^, which are believed to be the active sites for the aerobic oxidation of aldehydes to acids via radical chain reaction.^15^, ^16^, ^17^

Notably, when the reaction was performed in water using the RuCo_*x*_Ce_*y*_O_*z*_ the crotonaldehyde conversion and crotonic acid yield decreased substantially to ≈78 % and 9 %, respectively (Figure 1). Kinetic and mechanistic studies have shown that the oxidation of aliphatic aldehydes starts with the reaction of the carbonyl aldehyde group and the Co^3+^ sites to form acyl radicals, protons with the concomitant reduction of the cobalt sites to Co^2+^. The oxidation of the acyl radical with oxygen leads to an acylperoxy that abstracts a hydrogen from another aldehyde to yield a peracid and another acyl radical. This peracid undergoes protonation to carboxylic acid, water, and oxidized Co^3+^ sites.^15^, ^16^, ^18^ Due to the Lewis character of the Co centers it is possible that water competes with the acylperoxy radicals for the coordination sphere of the Co^3+^ cations. This competition in combination with the quenching of chain‐propagating radicals in aqueous phase, could explain the lower rates of reaction observed in water. Decreasing temperature from 100 °C to 80 and 60 °C did not improve the selectivity of the reaction in aqueous environments (Table 1). Instead, the selectivity to crotonic acid monotonically decreased as the temperature decreased, indicative of low activation barriers for the secondary reactions in the aqueous phase.

**Figure 1:**
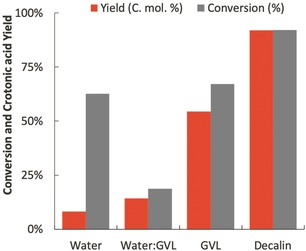
Crotonaldehyde conversion and crotonic acid yield obtained on different solvents during the aerobic oxidation reaction of crotonaldehyde at 38 bar of synthetic air and 80 °C using 200 mg of RuCo_*x*_Ce_*y*_O_*z*_ in decalin.

By changing the solvent to a dipolar aprotic organic solvent such as γ‐valerolactone (GVL) we observed similar conversion as that obtained in water (67 %), while the yield to crotonic acid increased to 54 % (Figure 1). The differences in yield in the two solvent environments was caused by the activation of acid catalyzed condensation reactions. In the aqueous phase crotonic acid can partly dissociate (crotonic acid p*K*_a_=4.69^19^ and water proton affinity 681.9 kJ mol^−1[20]^), lowering the pH of the reaction media and activating the vinylic bonds of the crotonaldehyde and crotonic acid. Furthermore, acid catalyzed aldol condensation of the crotonaldehyde could also be enhanced at low pH. For this reason, often oxidation reactions in aqueous environments are performed in alkaline media.^17^

The results of crotonaldehyde aerobic oxidation to crotonic acid using the ETS‐4 indicated that on decalin it is possible to achieve 58 % yield at 70 % conversion, which is similar to the results obtained on the Co_*x*_Ce_*y*_O_*z*_ catalyst. Notably, when the solvent was changed to acetic acid, the conversion and yield significantly increased resulting in quantitative production of crotonic acid (91 mol C %) in the presence of 50 mg of catalyst. This enhancement led to STY that were two orders of magnitude higher (11.3 mol g^−1^ h^−1^) than those obtained in the Ru Co_*x*_Ce_*y*_O_*z*_ catalyst in decalin (0.09 mol g^−1^ h^−1^). The higher yield and activity of this reaction could be attributed to the formation of peracetic acid. During aerobic oxidation the partially uncoordinated Ti cations can act as Lewis acid sites that activate the formation of peroxide species. These species can be selective oxidizing agents for the conversion of the crotonaldehyde to crotonic acid. Detailed batch reaction experiments were performed for several reaction times, using only 50 mg of ETS‐4 catalyst. About 84 % of conversion was obtained during the first three hours of reaction (Figure S1). At this point the rate of the reaction plateaued. Notably, at this point the selectivities achieved were 92–100 %.

Then, we studied the selective epoxidation of crotonic acid over Sharpless‐like catalysts based on tungsten oxide catalysts in the presence of diluted hydrogen peroxide as oxidant at 65 °C (step *d* in Scheme 1). We employed ^13^C‐NMR for product identification and HPLC for quantification. The results showed that before reaction only the crotonic acid can be detected by ^13^C‐NMR (Figure S2). After 3 h of reaction at pH 2.9 additional chemical shifts appeared in the ^13^C‐NMR spectra that corresponded to the formation of the epoxide (3‐methyloxirane‐2‐carboxylic acid) and α,β‐dihydroxy acid (2,3‐dihydroxybutanoic acid). At pH 2.9 the molar fraction of epoxide was lower (22 mol %) than that of the α,β‐dihydroxy acid (37 mol %) (Figure 2). The high yields to the di‐hydroxy acid (37 %) in the acidic environment can be ascribed to the hydrolysis of epoxide ring (Table S3). The exact mechanism of this reaction, step *e* in Scheme 1, is still under debate. Early work on kinetic isotope effects on the hydrolysis of ethylene oxide showed that there are two possible mechanisms (S_N_1 and S_N_2).^21^, ^22^, ^23^ In both mechanisms, the first step is the protonation of the epoxide oxygen in a fast equilibrium step.^21^, ^24^ The second step is either the decomposition of the conjugated acid followed by rapid reaction of the carbonium ion with water (S_N_1 mechanism) or the bimolecular substitution of the conjugated acid with water (S_N_2 mechanism).^25^ A similar strategy is applied industrially for the production of ethylene glycol from ethylene oxide, although at higher temperature (200 °C).^26^

**Figure 2:**
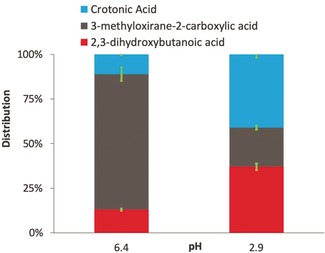
Molar distribution obtained at different initial pH (6.4 and 2.9) after 0.5 h of the epoxidation reaction of crotonic acid (0.255 mol L^−1^) at 65 °C and atmospheric pressure using 77 mg of WO_3_ catalyst synthesized by combustion. The volume of H_2_O_2_ was 230 μL of H_2_O_2_ at a concentration of 10 vol. %.

To reduce the rate of α,β‐dihydroxy acid formation we decided to increase the pH to 6.4 by adding KOH to the reaction mixture (Figure 2). The ^13^C‐NMR results indicated that the two main species present in the reaction mixture after 3 h of reaction were crotonic acid and the epoxide. At these conditions, the conversion of crotonic acid increased to 89 % and the yield of 3‐methyloxirane‐2‐carboxylic acid and 2,3‐dihydroxybutanoic acid were 76 and 22 %, respectively (see Table S3). Previous reports indicate that increasing the pH beyond 7 leads to faster rates of hydrolysis,^21^, ^24^ which in our case would have led to higher selectivity to 2,3‐dihydroxybutanoic acid. These results indicate that nearly neutral pH can effectively reduce the rate of epoxide hydrolysis increasing the selectivity towards the epoxide adduct.

The performance of the WO_3_ catalyst prepared using the combustion method was compared with commercial WO_3_ (Sigma–Aldrich), and SBA‐15 doped with WO_3_ prepared by incipient wetness impregnation (Figure 3). The molar distribution after 0.5 h of reaction indicates that with the WO_3_ combustion catalyst it was possible to obtain up to 54 mol % of the epoxide (3‐methyloxirane‐2‐carboxylic acid), while in the case of commercial WO_3_ and WO_3_‐SBA‐15 catalysts these values decreased to 15 and 7 %, respectively. With all the catalysts, formation of the α,β‐dihydroxy acid (2,3‐dihydroxybutanoic acid) was low due to the near neutral pH employed during reaction.

**Figure 3:**
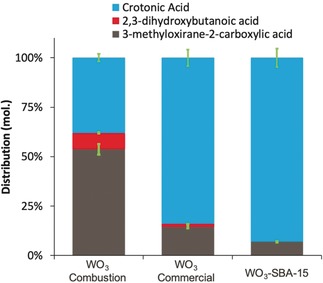
Molar distribution obtained after the reaction of crotonic acid (0.255 mol L^−1^) epoxidation at pH 6.4 and 65 °C and atmospheric pressure after 0.5 hours using 50 mg of catalysts and 230 μL of H_2_O_2_ at 10 vol. %.

Significantly lower conversions were obtained for the commercial WO_3_ and WO_3_‐SBA‐15, which only yielded 16 and 7 %, respectively (see Table S4). Here, it was observed that WO_3_ prepared using the combustion method achieved rates of 9 mol h^−1^ mol of WO_3_, while in the case of WO_3_ supported on SBA‐15 and commercial WO_3_ catalysts the rates were 6.1 and 2.3 mol h^−1^ mol of WO_3_, respectively. Diffuse reflectance UV‐vis characterization of the as‐prepared catalysts indicated that in the WO_3_ catalyst the UV‐visible absorption edge was shifted towards higher wavelengths in comparison to the WO_3_ commercial, which can be assigned to isolated tetrahedral and small oligomers WO_*x*_ (Figure S3). In contrast, the UV‐vis spectrum of WO_3_‐SBA‐15 catalyst showed an absorption maximum at 230 nm in combination with a superimposed shoulder at 260 nm, which can be assigned to isolated tetrahedral and small oligomers WO_*x*_. Previous kinetic and spectroscopic studies reported by C. Hammond et al.^28^ on epoxidation of cyclooctene in 1‐butanol using W‐Zn/SnO_2_ catalyst showed that the most active and stable species during epoxidation of cyclooctene was not the isolated W^iv^ species, but instead the polymeric and bulk WO_3_ phases. This could explain the higher activity of the WO_3_ prepared by combustion.

The HPLC analysis of the product mixture obtained from the homogeneous reduction of isolated 3‐methyloxirane‐2‐carboxylic acid in aqueous solution indicated that this reaction step can be readily accomplished with very high chemo‐selectivity (≈100 %) at 40 % conversion. These promising results served as basis for the evaluation of the second set of experiments using the un‐purified 3‐methyloxirane‐2‐carboxylic acid in the presence of 34 bar of H_2_ and 5 wt. % Ru/C catalyst. The reaction mixture was composed of crotonic acid, 3‐methyloxirane‐2‐carboxylic acid, and 2,3‐dihydroxybutanoic acid (Table 2).

**Table 2:** Concentration of reactant and products in the hydrogenation of crotonic acid epoxidation product on 50 mg of 5 wt. % Ru/C after 2 h of reaction at 180 °C, 34 bar of hydrogen and 800 rpm of agitation in a total volume of 100 mL.

Compound	Feed [mol L^−1^]	Product [mol L^−1^]	Conversion [%]	Yield [%]
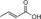	0.14	0	100	0
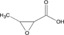	0.21	0	100	0
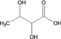	0.036	0.036	100	0
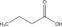	0	0.14	0	100
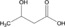	0	0.19	0	–

After reaction, the crotonic acid and 3‐methyloxirane‐2‐carboxylic acid were quantitatively hydrogenated towards butanoic acid and 3‐hydroxy‐butanoic acid, respectively. In contrast, the α,β‐dihydroxy acid (2,3‐dihydroxybutanoic acid) remained unreacted after the reaction. Notably, the chemoselectivity of the hydrogenation of 3‐methyloxirane‐2‐carboxylic acid towards 3‐hydroxy‐butanoic acid was 100 %.

In summary, the utilization of tandem oxidation, epoxidation, followed by epoxy‐ring activation by either hydrolysis or hydrogenation offers a flexible platform for the production of specialty chemical building blocks from biomass‐derived ethanol derivatives. This strategy allows for the selective conversion of bio‐ethanol to crotonic acid, 3‐hydroxy butanoic acid, and 2,3‐dihydroxyacid at high carbon yields (62, 21, and 29 %, respectively).

We have successfully demonstrated that using an intermediate step of epoxidation of the unsaturated acid it is possible to selectively produce β‐hydroxy acids and α,β‐dihydroxy acids, which are otherwise inaccessible via direct oxidation of the aldol‐adduct. We envision that the heterogeneous catalytic cascade approach that we have developed here can serve as a basis for the production of numerous high‐value chemical building blocks from bio‐ethanol. Furthermore, tuning the aldol condensation process to increase the yield towards C_6_ and C_8_ aldehydes could enable the production of long‐chain hydro‐acids for the production of long‐chain PHAs with enhanced mechanical properties.

## Conflict of interest

The authors declare no conflict of interest.

## Supporting information

As a service to our authors and readers, this journal provides supporting information supplied by the authors. Such materials are peer reviewed and may be re‐organized for online delivery, but are not copy‐edited or typeset. Technical support issues arising from supporting information (other than missing files) should be addressed to the authors.

Supplementary
