# Supplementary material for: Synthesis of α,β‐ and β‐Unsaturated Acids and Hydroxy Acids by Tandem Oxidation, Epoxidation, and Hydrolysis/Hydrogenation of Bioethanol Derivatives
Source: Angew Chem Int Ed Engl. 2020 Mar 11;59(19):7456–60. doi: 10.1002/anie.202002049 (PMC7217036; doi:10.1002/anie.202002049)
Supplement: Supplementary file 1 — Supplementary [file ANIE-59-7456-s001.pdf]

Angewandte Chemie
Eine Zeitschrift der Gesellschaft Deutscher Chemiker

Supporting Information

# Synthesis of α,β- and β-Unsaturated Acids and Hydroxy Acids by Tandem Oxidation, Epoxidation, and Hydrolysis/Hydrogenation of Bioethanol Derivatives

*Daniel Santhanaraj, Maria P. Ruiz, Mallik R. Komarneni, Tu Pham, Gengnan Li, Daniel E. Resasco, and Jimmy Faria**

anie_202002049_sm_miscellaneous_information.pdf

**Index**

1. Experimental details…3
1.1. Synthesis of oxidative dehydrogenation catalysts…3
1.1.1. $Cu/SiO_2$ and $Ni/SiO_2$…3
1.2. Aldol-condensation catalysts…3
1.3. Synthesis of oxidation catalysts…3
1.3.1. $Co_xCe_yO_z$…3
1.3.2. 5% $Ru/Co_xCe_yO_z$…3
1.3.3. Titanium silicate (ETS-4)…3
1.4. Epoxidation catalysts synthesis…4
1.4.1. Nanocrystalline $WO_3$…4
1.4.2. Tungsten incorporated on SBA-15 (W-SBA-15)…4
1.5. Catalysts testing…4
1.5.1. Oxidative dehydrogenation of ethanol…4
1.5.2. Aldol-condensation of acetaldehyde…4
1.5.3. Oxidation of crotonaldehyde to crotonic acid…5
1.5.4. Epoxidation of crotonic acid…5
1.5.5. Hydrogenation of 3-methyloxirane-2-carboxylic acid…5
1.5.6. Diffuse reflectance UV-vis characterization…5
2. Catalytic testing…7
2.1. Selective Oxidation of ethanol to acetaldehyde in vapor phase…7
2.2. Aldol-condensation of acetaldehyde to crotonaldehyde…7
2.3. Oxidation of crotonaldehyde to crotonic acid…9
2.4. Epoxidation of crotonic acid…9
2.4.1. Effect of pH…9
2.4.2. $WO_3$ Catalyst screening…11
2.4.3. Solvent screening…12
2.4.4. Catalyst stability in aqueous environments…12
2.5. Hydrogenation of epoxy acid…14
2.5.1. Two step hydrogenation using Pd/C followed by $NaBH_4$ treatment…14
2.5.2. Epoxide extraction followed by hydrogenation by $NaBH_4$…15
2.5.3. Hydrogenation on 5 wt. % Ru/C…16
3. Product characterization by $^1H$- and $^{13}C$- NMR…17
4. Preliminary technoeconomic analysis…18
4.1. Conceptual process design…19
4.2. Distillation resistance…19
5. References…23

## 1. Experimental

### 1.1. Oxidative dehydrogenation catalysts

The conversion of ethanol to acetaldehyde was tested using oxidative dehydrogenation catalysts based on Cu and Ni supported on $SiO_2$.

**1.1.1. Cu/$SiO_2$ and Ni/$SiO_2$:** These catalysts were prepared by incipient wetness impregnation (IWI) of the support ($SiO_2$, Hisil 210) with an aqueous solution of the Cu ($Cu(NO_3)_2$) and Ni ($Ni(NO_3)_2$) metal precursors obtained from Fluka (99% purity). To achieve the desired metal loading of 5 wt. % for Ni and 10 wt. % fo Cu metal salt aqueous solutions with the appropriate concentrations were impregnated onto the silica support, using a solid/liquid ratio of 1 g/cm$^3$. Upon impregnation, the catalysts were dried at room temperature overnight followed by 12 h of drying in an oven at 120 °C. Finally, the oven-dried catalysts were calcined for 4 h at 400 °C in a flow reactor using a linear heating ramp of 10 °C/min, under a flow of pure air at 100 mL/min.

### 1.2. Aldol-condensation catalysts

Magnesium oxide (MgO, .99%) was purchased from Sigma–Aldrich and used as received. The parent faujasite KNaX zeolite (Si/Al=1.0) was synthesized by a conventional hydrothermal method. The Al-Beta zeolite (Si:Al mole ratio of 12.5) was purchased from Zeolyst (ZEO041 (BEA) CP-806E-22). The hydrotalcite (DHT-6) with Mg/Al ratio of 3 was purchased from Kyowa Chemical Industry, Japan. The 7.5 wt. % B doped MgO samples were prepared by the thermal decomposition (citrate–nitrate combustion) method,[1] using $Mg(NO_3)_2$ (Sigma–Aldrich, 99%) and $B_2O_3$ (Alfa Aesar, 99.99%) as precursors. $Mg(NO_3)_2$ and $B_2O_3$ corresponding to the desired composition were dissolved in deionized water. As soon as the solution became clear, citric acid was added in a proportion of 1.2 moles of the acid per mole of metal atom. The mixture was then heated on a hot plate (at 140 °C) for 1 h to evaporate the water. After that, it was quickly transferred and combusted inside an oven preheated to 550 ºC. The ashes obtained after combustion were calcined at 550 °C for 12 h in air to eliminate the carbonaceous residues. The resulting material was then ground into a fine powder for use. The MgO-KNaX faujasite was prepared as reported in previous studies.[2,3] $Mg^{2+}$ ions were introduced into the zeolite by the ion-exchange method, in which the zeolite sample (2 g) was contacted with an aqueous solution of $Mg(NO_3)_2$ (100 mL) at room temperature for 24 h. To adjust the content of $Mg^{2+}$ ions in the zeolite, the concentration of the solution of $Mg(NO_3)_2$ was varied from 0.1 to 1 M. After filtration and drying overnight at 120 °C, the resulting $Mg^{2+}$-containing product was added to a solution of KOH (100 mL) to precipitate the $Mg^{2+}$ ions in the $Mg(OH)_2$ form. In this step, the concentration of the alkaline solution was the same as that of the $Mg(NO_3)_2$ solution. After stirring for 20 min, the sample was recovered by filtration and washing with deionized water to reach a pH value close to 7. The solid sample obtained was then dried at 120 °C for 6 h, followed by calcination at 400 °C for 1 h, to obtain the MgO-modified faujasite product.

### 1.3. Oxidation catalysts synthesis

Three different catalysts were synthesized to study the conversion of crotonaldehyde to crotonic acid: $Co_xCe_yO_z$, 5 wt.% Ru/ $Co_xCe_yO_z$ and titanium silicate (ETS-4).

**1.3.1. $Co_xCe_yO_z$.** The catalyst was synthesized by the precipitation method described elsewhere.[4] Initially, a solution of $Co(NO_3)_2.6H_2O$ (Sigma-Aldrich, 99.999 %) and $Ce(NO_3)_3.6H_2O$ (Sigma-Aldrich, 99.999 %) was prepared in a molar ratio 0.1:0.05 in deionized (DI) water. An aqueous solution of $Na_2CO_3$ and NaOH prepared in a molar ratio 0.13:0.46 was added slowly to the aqueous solution of nitrates with continuous stirring at room temperature to form a precipitate of the corresponding metal hydroxides. The dispersion was agitated for 2 hours at atmospheric conditions. The metal hydroxide slurry was then filtrated using a 5 µm filter paper and washed thoroughly with DI water. The slurry was further dried at 100 °C for 12 h and calcined at 450 °C for 4 h.

**1.3.2. 5% Ru/$Co_xCe_yO_z$.** The catalyst was synthesized following a similar precipitation method to the one described above. A solution of $RuCl_3.H_2O$ (Sigma-Aldrich, 99.98%), $Co(NO_3)_2.6H_2O$ (Sigma-Aldrich, 99.999 %) and $Ce(NO_3)_3.6H_2O$ (Sigma-Aldrich, 99.999 %) was prepared in a molar ratio 0.015:0.1:0.05 in DI water. An aqueous solution of $Na_2CO_3$ and NaOH prepared in a molar ratio 0.13:0.46 was added slowly to the aqueous solution of nitrates with continuous stirring at room temperature to form a precipitate of the corresponding metal hydroxides. The dispersion was agitated for 2 hours at atmospheric conditions. The dark brown slurry was then filtrated using a 5 µm filter paper and washed thoroughly with DI water. The slurry was further dried at 100 °C for 12 h and calcined at 450 °C for 4 h.

**1.3.3. Titanium silicate (ETS-4).** A solution of sodium metasilicate pentahydrate ($Na_2SiO_3.5H_2O$, Sigma-Aldrich, >95%) and sodium hydroxide (NaOH, Sigma-Aldrich, 99.99%) was prepared in DI water to make. A solution of titanium trichloride ($TiCl_3$, Sigma-Aldrich, 99.99%) and potassium fluoride

anhydrous (KF, Sigma-Aldrich, 99.999%) was added in continuous stirring. Once the agitation was stopped the titanium-silicalate gel was filtrated using a 5 µm filtration paper and thoroughly washed with DI water. The gel was later dried at 100 °C for 12 h and calcined at 450 °C for 4 h.

### 1.4. Epoxidation catalysts synthesis

For the epoxidation reaction, two catalysts were synthesized: nanocrystalline $WO_3$ and tungsten on SBA-15.

**1.4.1. Nanocrystalline $WO_3$.** Nanocrystalline $WO_3$ was prepared by combustion method using ammonium metatungstate hydrate ($H_{26}N_6O_4W_{12}.xH_2O$, 99.99%, Alfa Aesar) and citric acid ($HOC(COOH)(CH_2COOH)_2$, 99.5%, Alfa Aesar). In a typical procedure to synthesize nanocrystalline $WO_3$, 2 g of ammonium metatungstate hydrate and 1.87 g of citric acid (1.2 moles per 1 mole of W atoms) were dissolved in 6 mL of HPLC grade water. The mixture was stirred well for 10 minutes and then transferred to a crucible. The crucible was then placed in an oven preheated to 550 °C. After 15 minutes, the crucible was removed from the oven and allowed to cool to room temperature. $WO_3$ formed by combustion was fluffy in appearance. The obtained $WO_3$ catalyst was then ground to a fine powder and calcined stepwise in 100 cc/min air as follows: 1 °C/min up to 250 °C and held for 2 h, 3 °C/min up to 550 °C and held for 12 h. The calcined $WO_3$ catalyst was cooled down to room temperature in the flow of air.

**1.4.2. Tungsten incorporated on SBA-15 (W-SBA-15).** The tungsten incorporated mesoporous SBA-15 (Si/W=20) samples were synthesized hydrothermally using Sodium tungstate as a tungsten source. The modified synthesis procedure was as follows: 4.64 g of triblock copolymer poly (ethylene glycol)–block-poly (propylene glycol)-block-poly(ethylene glycol)-(Pluronic P123, molecular weight = 5,800, EO20 PO70 EO20) was dissolved in 30.0 g of deionized water and stirred for 4 h. 9.0 g of tetraethyl orthosilicate and the 0.73 g of sodium tungstate were added directly to the clear solution. Then, 70 mL of 0.28 M HCl were added to the above solution. The gel was stirred for 24 h and then maintained at 100 °C for another 48 h. The resultant precipitate was washed thoroughly with distilled water for several times and finally dried at 70 °C for 12 h. The final solid was then calcined in air at 550 °C for 6 h.

### 1.5. Catalysts testing

**1.5.1. Oxidative dehydrogenation of ethanol.** The catalysts were pelletized, crushed and sieved to the range 250–425 µm and placed at the isothermal region of the ¼ inch quartz reactor between two layers of glass beads and quartz wool. The amount of catalysts loaded in the reactor ranged between 40 mg, the air flow rate was adjusted to reach 0.5 h. The catalyst was pre-reduced in $H_2$ flow (60 mL/min, Airgas, 99.99%) for 1 h at 350 °C for 10 wt. % $Cu/SiO_2$ and 450 °C for $Ni/SiO_2$. After this pre-treatment, the catalyst was cooled down to the selected reaction temperature (250 °C) under air or He flow (100 $mL*min^{-1}$) mixed with an ethanol flow rate of 0.25 $mL*h^{-1}$. The products were analyzed by an on-line on-gas chromatograph (Agilent model 6890) equipped with a HP-5 capillary column and a FID detector. The carbon balance was above 95% in every experiment. The conversion of ethanol and product selectivity ($S_i$) were calculated and defined as follows:

$$\boldsymbol{X_{ethanol}}\ (\%) = \frac{\boldsymbol{mol\ of\ ethanol\ in\ the\ feed - mols\ of\ ethanol\ in\ the\ product}}{\boldsymbol{mol\ of\ ethanol\ fed}} * \mathbf{100}\ \textbf{Eq. 1}$$

$$\boldsymbol{S_i}\ (\%) = \frac{\boldsymbol{mol\ of\ product_i}}{\boldsymbol{mol\ of\ ethanol\ consumed}} * \mathbf{100}\ \textbf{Eq. 2}$$

**1.5.2. Aldol-condensation of acetaldehyde.** These experiments were conducted following previously reported methodology by our group.[5] In a typical experiment, a specific mass of catalyst was mixed with 2 mL of acetaldehyde (100%, Pharmco AAPER) and 20 mL of ethanol (100%, Pharmco AAPER) in a 50 mL stainless-steel autoclave batch reactor (Parr Corporation). The reactor was sealed, purged and pressurized in nitrogen to 20.7 bar and heated up to the desired reaction temperature (180 ºC) under 750 rpm of agitation. The reaction products were filtered and analysed using gas chromatography (GC). Product identification was conducted on a GC–MS (Shimadzu QP2010S) equipped with a ZB-1701 column, 60.0 mV0.25 mm nominal. GC–FID (Agilent 7890B) with a capillary column of poly(ethylene glycol) (ZB-WAX) of 60.0 mV0.25 mmV0.25 mm nominal was employed for

quantification, using the corresponding chemical standards to obtain response factors. All the yields were calculated in carbon molar base. The detectable products consisted of C4's (crotonaldehyde, crotyl alcohol, 3-buten-1-ol, butanal, butanol), 1,1-diethoxyethane, ethyl acetate, mixed C6 (2,4-hexadienal), and mixed C8 (2,4,6-octatrienal, meta- or ortho-tolualdehyde, ethyl-3-hexenoate, and ethylhexanoate).

**1.5.3. Oxidation of crotonaldehyde to crotonic acid.** Liquid phase partial oxidation of crotonaldehyde (Sigma Aldrich, 99%) was carried out in a 50 mL stainless steel autoclave batch reactor (Parr Instrument Co.), equipped with a variable speed impeller, heater, temperature and pressure controllers. In a typical experiment, the powder catalyst sample (e.g. 200 mg) was dispersed in 20 mL of solvent in the Parr reactor vessel. The reactor was sealed, purged, and pressurized with air to 38 bar. It was then heated to the reaction temperature of 80 °C. The reaction was carried out at high stirring speed to avoid external mass transfer limitations. At the end of the reaction the stirring was stopped, and the reactor was cooled down to room temperature. After cooling, the liquid products were filtered and analyzed by gas chromatography with flame ionization detection. Gas chromatography-mass spectrometry (GC-MS Shimadzu) was used for product identification.

**1.5.4. Epoxidation of crotonic acid.** Crotonic acid epoxidation was carried over $WO_3$ (commercial and the one produced by the combustion process) and SBA-15 at two different pH values: 2.4 (stock solution) and 6.4. In a typical experiment, 0.35 M aqueous solution of crotonic acid was neutralized to pH 6.4 by adding 1N KOH to obtain 0.26 M solution. 77 mg of the catalyst was added to 6 mL of 0.255 M crotonic acid solution at pH 6.4 in a 22 mL glass vial. The vial was capped and placed in an oil bath at 65 °C and the reactant contents were stirred for 10 minutes at 1200 rpm. After 10 minutes, 0.23 mL of 10.6 M $H_2O_2$ was added. The reaction was carried out at 65 °C for 3 h at 1200 rpm. After the reaction, the catalyst was filtered through a 0.2 µm syringe filter. The reaction products were then analyzed by high-performance liquid chromatography (HPLC) equipped with Aminex HPX-87H column (Waters Company) using 5 mM $H_2SO_4$ as the mobile phase. $^1$H- and $^{13}$C-NMR techniques were used to confirm the product distribution in the epoxidation reaction. The results of the product identification of the reaction of crotonic acid to 3-methyloxirane-2-carboxylic acid and the standard molecule crotonic acid are presented in the Supplementary Information. This step was critical as it was possible to identify that the resonance peaks at 3.2 and 1.2 ppm corresponded to the H- close to the carbonyl carbon and the -$CH_3$ of the epoxide (3-methyloxirane-2-carboxylic acid), which are not observed in the H-NMR of the pure crotonic acid.

**1.5.5. Hydrogenation of 3-methyloxirane-2-carboxylic acid.** Two different strategies were employed for the hydrogenation of the epoxy acid. The first one consisted in a non-catalytic homogeneous reduction using $NaBH_4$ as reducing agent and purified epoxy acid as reaction substrate, while in the second one the un-purified epoxy acid product mixture was reduced using molecular hydrogen on a 5 wt.% Ru/C catalyst. For the homogeneous reduction of the epoxy acid the product mixture containing 2,3-dihydroxybutanoic acid, 3-methyloxirane-2-carboxylic acid, and unreacted crotonic acid was transferred to a 200 mL beaker and neutralized with dilute sulfuric acid and supersaturated with ammonium sulfate salt. The free epoxy acid product was extracted with diethyl ether and the solvent was evaporated to dryness to obtain crude solid product. The resulting mixture was analyzed by HPLC to confirm the purity of the product before reaction. Then, the dried solid product was diluted with water and hydrogenated with 50 mg sodium borohydride at room temperature. The reaction products were then analyzed by high-performance liquid chromatography equipped with Aminex HPX-87H column (Waters Company) using 5 mM $H_2SO_4$ as the mobile phase.

Additional hydrogenation experiments were performed using commercial 5 wt.% Ru/C catalyst (Sigma-Aldrich) using a stainless-steel reactor of 180 $cm^3$ from Parr Instrument Co. In a typical experiment, the epoxidation product mixture obtained after 3 h of reaction at 65 °C and pH of 6.4 using the $WO_3$ catalyst synthesized by combustion was employed as the starting feedstock for the hydrogenation reaction. Initially, 0.5 g catalyst sample (5 wt.% Ru/C from Sigma-Aldrich) was added to 75 mL of water in the reactor and the catalyst was pre-reduced in hydrogen at 250 °C and 5 bar for 30 min with stirring. After the catalyst pre-reduction, 25 mL of epoxy acid reaction mixture containing 2,3-dihydroxybutanoic acid, 3-methyloxirane-2-carboxylic acid, and unreacted crotonic acid was introduced into the reactor. The reaction was performed at 150 °C for 3 h and at 500 psi of hydrogen with a stirring speed of 800 rpm to prevent external mass diffusion limitations. After completion of the reaction, the catalyst was separated by filtration and the products were identified by $^1$H- and $^{13}$C-NMR (Bruker Avance-400). Quantitative determination of the reaction products was carried out by HPLC using an Aminex HPX-87H column (Waters Company) and 5 mM $H_2SO_4$ as the mobile phase.

**1.5.6. Diffuse reflectance UV-vis characterization.** Diffuse reflectance UV-vis characterization was performed on the fresh catalyst samples using a Shimadzu UV 2450 dual beam spectrophotometer.

The solid samples were analysed by diffuse reflectance employing the dual beam to minimize noise between the baseline and the sample.

## 2. Catalytic testing

### 2.1. Selective Oxidation of ethanol to acetaldehyde in vapor phase

Ethanol oxidative dehydrogenation to acetaldehyde is shown in **Scheme S1**.

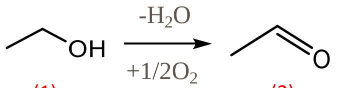


(1) (2)

**Scheme S1.** Dehydrogenation of ethanol in oxidative environment.

**Table S1** shows the conversion and selectivity of the Ni and Cu based catalysts obtained after oxidative dehydrogenation. The results indicate that the catalytic activity of 10 wt. % Cu/$SiO_2$ catalyst was higher than that observed on the 5 wt. % Ni/$SiO_2$ catalysts with values of 0.67 ± 0.06 and 0.46 ± 0.02 gEtOH*gcat$^{-1}$*h$^{-1}$, respectively. These difference in catalytic activity could be either caused by the differences in total metal loading on the Cu/$SiO_2$ catalyst compared to the Ni/$SiO_2$ or the higher oxophilicity of Cu metal surface that could enhance $\eta^1$-CO binding via the oxygen of the carbonyl carbon.[6] Regardless of the metal employed we observed remarkable selectivity towards the corresponding aldehyde. Achieving high-atom efficiencies on oxidative dehydrogenation reactions is a rather challenging task as the first formed products are typically more prone to undergo further oxidation and undesired coupling products.[7] For this reason, many industrial processes that involve selective oxidation reactions are performed at low levels of conversion and high-spatial velocities to control the heat evolved during reaction and the reaction selectivity. For the purpose of this study, it is imperative to achieve high-selectivity to avoid undesirable losses of expensive bio-ethanol feedstock via sequential oxidation. Furthermore, we observed that in order to perform the C-C coupling selectively towards the $C_4$'s aldehydes it is important to maintain the aldehyde to alcohol volume ratio 1:10 to avoid secondary reactions. This would allow us to directly feed the products form the oxidative dehydrogenation unit into the aldol-condensation reactor.

**Table S1.** Conversion and selectivity of the ethanol dehydrogenation reaction under oxidative and non-oxidative environments at 250 °C, 1 bar, catalyst to feed ratio of 0.15 h.

| Catalyst | Gas phase | Conversion (%) | Selectivity (%) | Activity (g EtOH*gcat$^{-1}$*h$^{-1}$) |
|---|---|---|---|---|
| 10 wt. % Cu/$SiO_2$ | air | 14 (1) | 100 | 0.67 (0.06) |
| 5 wt. % Ni/$SiO_2$ | air | 9 (0.4) | 100 | 0.46 (0.02) |
| 10 wt. % Cu/$SiO_2$ | He | 4 (0.07) | 100 | 0.204 (0.003) |
| 5 wt. % Ni/$SiO_2$ | He | 0 | 0 | 0 |

**Note:** The carbon mass balance closure in all these experiments was close to 99 %. The values in parenthesis is the standard deviation between repetitions of the experiment determined after 0.5 and 1 h of reaction. The catalyst deactivation observed in these experiments was negligible. EtOH denotes Ethanol. Synthetic air flow of 100 mL*min$^{-1}$, ethanol flow rate of 0.25 mL*h$^{-1}$ and 40 mg of catalyst.

### 2.2. Aldol-condensation of acetaldehyde to crotonaldehyde

As shown in **Scheme S2** the aldol-condensation reaction of acetaldehyde leads to the formation of 3-hydroxybutanal, which quickly dehydrates to form crotonaldehyde. The conversion of the resulting ethanol and acetaldehyde mixture to the corresponding $C_4$'s unsaturated aldehydes were tested using a variety of catalysts (see **Table S2**) at 180 °C and 20 bar. Notably, the most active catalysts were the

B-doped magnesium oxide (7.5 wt. % B-MgO) and Al-Beta catalysts with productivities of high as 24 mmol*$g_{cat}^{-1}$*h at 180°C at 50-60% conversion.

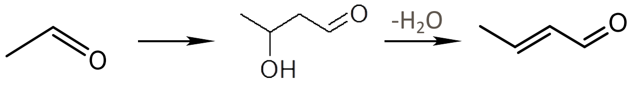


**Scheme S2:** Aldol-condensation of acetaldehyde (a) to 3-hydroxy-butanal (b) followed by dehydration to crotonaldehyde (d).

In contrast, the zeolitic KNaX catalyst showed the lowest catalytic productivity and activity towards the formation of $C_4$ products with values of 0.2 mmol*$g_{cat}^{-1}$*$h^{-1}$ at 180 °C. By changing from zeolitic to mixed oxide material derived from Mg-Al hydrotalcite it was possible to achieve a $C_4$ productivity of c.a. 8 mmol*$g_{cat}^{-1}$*$min^{-1}$. This productivity was higher than that of MgO, KNaX-MgO, and KNaX catalysts at 180 °C and the same level of conversion (50-60 %). On the MgO and KNa-MgO catalysts the productivity obtained was similar after 3 h of reaction (e.g. 4 to 5 mmol of $C_4$*$g_{cat}^{-1}$*$h^{-1}$**)**. The main issue with these catalysts was the low selectivity towards the $C_4$ products, due to base-catalyzed aldol-condensation to oligomers. On the KNaX catalyst the selectivity towards acid-catalyzed reactions (e.g. acetalization) combined with lower catalytic activity decreased the productivity of $C_4$s significantly.-In summary, the more basic Mg-Al hydrotalcite and 7.5 wt. % B-MgO catalysts showed the highest activities and $C_4$ productivities. Notably, when the conversion level reached 80-90% a significant drop in selectivity was observed, leading to lower productivities (c.a. 15 mmol of $C_4$*$g_{cat}^{-1}$*$h^{-1}$). This trade-off in selectivity and conversion is not unique to our system as consecutive C-C coupling reaction rapidly decrease the yield. In our previous study of the B-MgO system we found that on the B-MgO catalyst containing 7.5 wt.% of boron it was possible to achieve 75 % yield of $C_4$ products at 250 ºC. We attributed this to the optimal balance of acid–base properties, meso-/macro-porosity, and surface area. The material showed minimal loss in catalytic activity after four cycles of reaction. These losses in activity could be reversed by calcination of the spent catalyst at 500 °C.[8]

**Table S2.** Catalytic screening for the aldol-condensation of acetaldehyde in ethanol solution in a batch reactor operating at 700 rpm in nitrogen. Catalyst mass varied from 0.4 to 0.2 g of catalyst and the reaction time was 3 to 5 hours. Productivity was measured at the end of the reaction.

| **Catalyst** | **Aldehyde: Alcohol Vol. ratio** | **Temp. (℃)** | **Conversion (%)** | **Selectivity (%)** | **Productivity (mmol of $C_4$*$g_{cat}^{-1}$*$h^{-1}$)** |
|---|---|---|---|---|---|
| Al-Beta | 1:10 | 180 | 54 | 95 | 23.4 |
| Mg-Al hydrotalcite | 1:10 | 180 | 48 | 42 | 8.3 |
| 7.5 wt. % B-MgO | 1:10 | 180 | 56 | 93 | 24.2 |
| Al-Beta | 1:10 | 180 | 85 | 79 | 15.2 |
| Mg-Al hydrotalcite | 1:10 | 180 | 85 | 26 | 5.7 |
| 7.5 wt. % B-MgO | 1:10 | 180 | 87 | 67 | 13.2 |
| KNaX& | 1:10 | 180 | 2 | 100 | 0.2 |
| MgO-KNaX& | 1:10 | 180 | 51 | 84 | 4.8 |
| MgO* | 1:10 | 180 | 68 | 54 | 4.1 |

**Note:** Experiments were conducted at 20 bar in a batch reactor of 50 mL. The carbon mass balance in these experiments was above 90 wt. %. (*) Reproduced from reference [5]. (&) Reproduced from reference [3].

## 2.3. Oxidation of crotonaldehyde to crotonic acid

The reaction of oxidation of crotonaldehyde to crotonic acid using molecular oxygen is shown in **Scheme S3**.

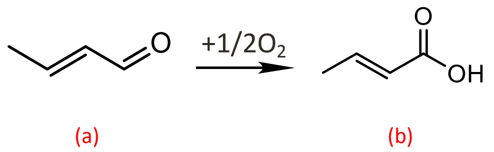


**Scheme S3.** Aerobic oxidation of crotonaldehyde (a) to crotonic acid (b).

When conducting catalytic reactions in aqueous media (see **Fig. 1**) the polar solvent may affect the catalyst performance by several possible phenomena that include; activation of undesired reactions, the dissolution of the active site into the bulk water phase (lixiviation), the destruction of the crystalline structure, and combinations of thereof.[9,10] However, these reactions were performed at mild (80 °C), in which the rate of hydrothermal deactivation is not very significant.[10–12] Instead, one could argue that the lower activity is cause by an inhibition effect.

Next, we studied the ETS-4 catalyst for the aerobic oxidation of crotonaldehyde in acetic acid media as a function of time (**Fig. S1**). The results indicate that on this catalyst system c.a. 84 % of conversion was achieved after only 3 h of reaction when using 50 mg of catalyst. At this level of conversion, the selectivity towards crotonic acid was 99.4 %.

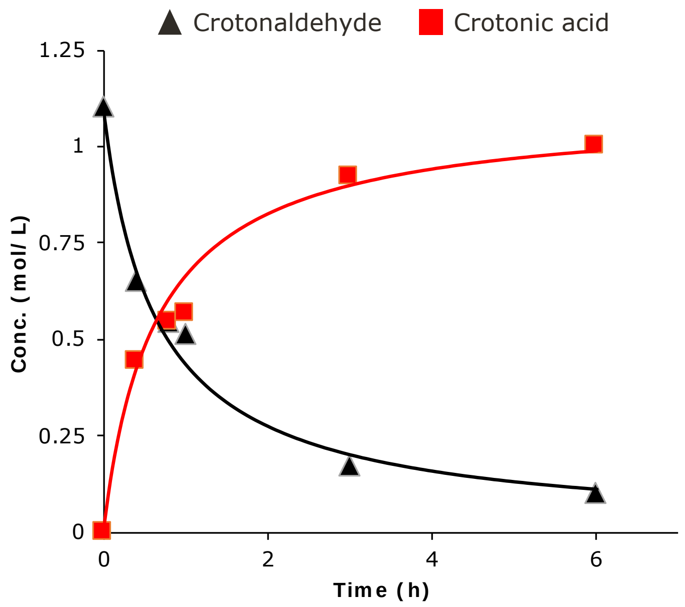


**Figure S1**: Evolution of crotonaldehyde oxidation to crotonic acid with the catalyst and reaction time (mg/h) in 38 bar of air over 50 mg of ETS-4 mixed oxides catalyst at 80 °C in acetic acid.

## 2.4. Epoxidation of crotonic acid

**2.4.1. Effect of pH.** The crotonic acid epoxidation was evaluated in acidic (pH 2.9) and near neutral (pH 6.4) media using the $WO_3$ catalyst prepared by the combustion method. The results showed that before reaction only the crotonic acid can be detected by $C^{13}$-NMR (**Fig. S2**). After 3h of reaction at pH 2.9 additional chemical shifts appeared in the $C^{13}$-NMR spectra that corresponded to the formation of the epoxide (3-methyloxirane-2-carboxylic acid) and α,β-dihydroxy acid (2,3-dihydroxybutanoic acid).

When the reaction was performed at pH of 6.4 we observed a significant increase in the formation of the epoxide at the expense of reducing the yield of α,β-dihydroxy acid (**Fig. S2 c-d**). The $C^{13}$-NMR results indicated that the two main species present in the reaction mixture after 3 h of reaction were crotonic acid and the epoxide.

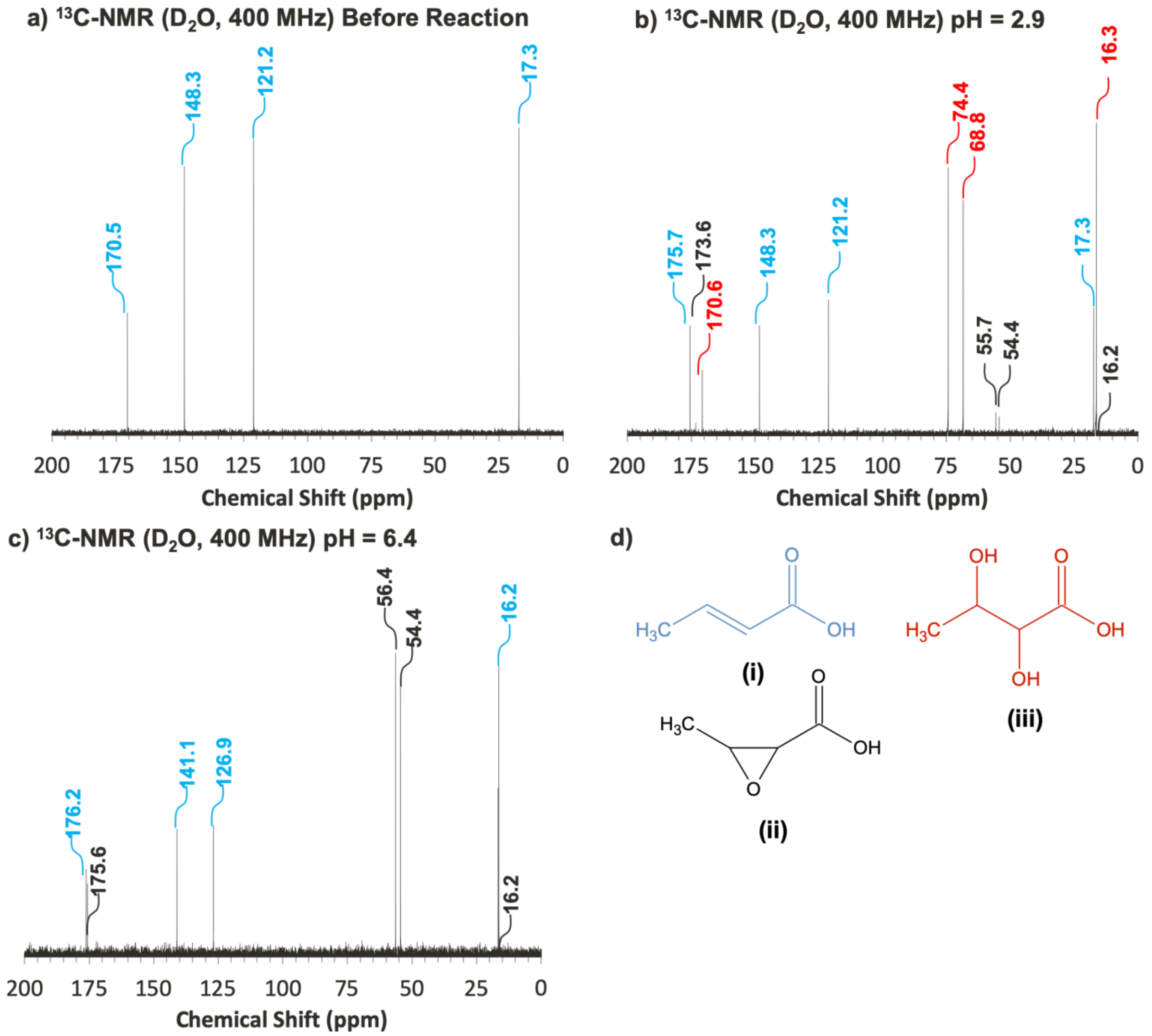


**Figure S2.** Epoxidation of crotonic acid at different initial pH values. $^{13}$C-NMR ($D_2O$, 400 MHz) spectra of crotonic acid before reaction (a), after reaction at pH 2.9 (b) and pH 6.4 (c) using KOH as pH-modifier. The following molecules were identified in the NMR; (i) crotonic acid, (ii) 3-methyloxirane-2-carboxylic acid, and (iii) 2,3-dihydroxybutanoic acid (d).

The quantification by HPLC indicate that in acid media the reaction rate was 0.91 $mol*h^{-1}*molWO_3^{-1}$, while at pH 6.4 the activity obtained was 1.37 $mol*h^{-1}*molWO_3^{-1}$ (**Table S3**). The yield towards the epoxy acid product in the near neutral media was 76 % and 13 % for the α,β-dihydroxy acid at elevated conversion of the crotonic acid (89 %). In contrast, at low pH the yield to the hydrolyzed epoxide (2,3-dihydroxybutanoic acid) increased to 37% and the conversion decreased to 59 %.

**Table S3:** Epoxidation of crotonic acid at different initial pH values after 3 h of reaction at 65 °C and 1 bar using 230 µL of $H_2O_2$ at 10 % (v/v) in the presence 77 mg of $WO_3$ (catalyst synthesized by combustion) and 0.25 mol/L of crotonic acid.

| pH | STY ($mol*h^{-1}*molWO_3^{-1}$) | Conversion (%) | Yield (mol. %) | |
|---|---|---|---|---|
| | | | 3-methyloxirane-2-carboxylic acid | 2,3-dihydroxybutanoic acid |
| 6.4 | 1.37 | 89 | 76 | 13 |
| 2.9 | 0.91 | 59 | 22 | 37 |

**Note:** STY refers to the site time yield calculated as the moles of product per mol of catalyst per unit of time.

**2.4.2. $WO_3$ Catalyst screening.** We explored the catalytic epoxidation of crotonic acid using three different catalytic materials based on tungsten oxide (**Table S4**) that included; $WO_3$-combustion, $WO_3$-Commercial, and $WO_3$-SBA-15. The results at buffered pH of 6.4 indicated that in the case of the catalyst synthesized by the combustion method the TOF reached values as high as 8.8 $mol*h^{-1}*mol\ WO_3^{-1}$, while the $WO_3$-SBA-15 and $WO_3$ commercial reached values of 2.3 and 6.1 $mol*h^{-1}*mol\ WO_3^{-1}$, respectively. The yield for the 3-methyloxirane-2-carboxylic acid varied on the three different catalysts from 54 to 100 %.

**Table S4.** Catalytic activity and selectivity of the different $WO_3$ catalysts obtained after 0.5 h of reaction of crotonic acid (0.255 mol/L) epoxidation at pH 6.4 and 65 °C and atmospheric pressure using 50 mg of catalysts and 230 µL of $H_2O_2$ at 10 vol. %.

| Catalysts | STY ($mol*h^{-1}*mol_{WO3}^{-1}$) | Conversion (%) | Yield ( mol. %) | |
|---|---|---|---|---|
| | | | 3-methyloxirane-2-carboxylic acid | 2,3-dihydroxybutanoic acid |
| $WO_3$ Combustion | 8.8 | 62 | 54 | 8 |
| $WO_3$ Commercial | 2.3 | 16 | 15 | 1 |
| $WO_3$-SBA-15 | 6.1 | 7 | 7 | 0 |

**Note:** STY refers to the site time yield calculated as the moles of product per mol of catalyst per unit of time.

UV-vis diffuse reflectance spectroscopy of the different catalysts is shown in **Figure S3**. The results were obtained on the as prepared catalysts. The spectra of the commercial and combustion $WO_3$ catalysts were very similar to what has been reported previously for $WO_3$.[13–17] These samples had a strong photo-absorption from the UV to visible light region with an absorption edge at approximately 460 nm that is attributed to the interband transition of bulk $WO_3$ (bandgap of 2.7-2.8 eV).[18] In the $WO_3$ combustion catalyst the UV-visible absorption edge is clearly shifted towards higher wavelengths. This can be attributed to the presence of oligomeric species. In contrast, in the UV-vis spectrum of $WO_3$-SBA-15 catalyst an absorption maximum was observed at 230 nm in combination with a superimposed shoulder at c.a. 260 nm, which can be assigned to isolated tetrahedral and small oligomers WOx. Previous work from R. Webber[19] and E. Iglesia et al.[20] on UV-vis characterization of W and Mo metal oxides demonstrates that the low energy absorption edge is shifted to higher wavelengths as a function of the nuclearity of the metal oxide species for domain sizes ranging from 1 to 10 nm. Thus, it is likely that in the $WO_3$-SBA-15 catalyst the majority of the tungsten is present in small clusters of $WO_x$, resembling that of the standard sodium tungstate ($Na_2WO_4*2H_2O$). This will indicate that the catalysts containing polymeric and bulk $WO_3$ have higher activity than those containing isolated $WO_x$ species. These results are in line with previous kinetic and spectroscopic studies reported by C. Hammond et al.[21] on epoxidation of cyclooctene in 1-butanol using W-Zn/$SnO_2$ catalyst. The authors demonstrated the most active and stable phase during cyclooctene epoxidation was not the isolated $W^{iv}$ species, but instead the polymeric and bulk $WO_3$ phases were determining the catalyst performance.

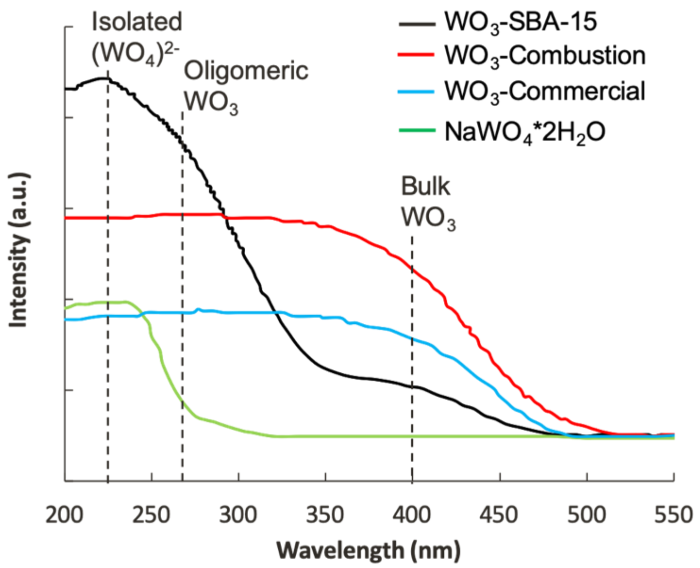


**Figure S3.** DRUV-vis spectroscopy of the $WO_3$ commercial, $WO_3$ combustion, $WO_3$-SBA-15 catalysts, and a standard $NaWO_4$.

**2.4.3. Solvent screening.** When the reaction was performed in organic solvents the activity of the $WO_3$ prepared by combustion drastically decreased (**Table S5**). Notably, the activity on dimethylsulfoxide, dichloromethane, γ-valerolactone, and methanol was negligible. In acetonitrile and water, the activity it was possible to observe epoxidation activity with conversions of 3 and 59 %, respectively.

**Table S5:** Conversion and reaction rates obtained in different solvents after epoxidation reaction of crotonic acid at 65 °C and atmospheric pressure using 230 µL of $H_2O_2$ at 10 vol. % and 0.05 g of $WO_3$ commercial catalyst (no base was added in these experiments) after 3 h of reaction.

| Solvent | Mass of Catalyst (g) | Crotonic acid (mol/L) | Conversion (%) | STY (mol*$h^{-1}$*mol$WO_3^{-1}$) |
|---|---|---|---|---|
| Water* | 0.077 | 0.2552 | 59 | 0.91 |
| Acetonitrile | 0.05 | 0.3356 | 3 | 0.09 |
| Dimethylsulfoxide | 0.05 | 0.3356 | 0 | 0 |
| Dichloromethane | 0.05 | 0.3356 | 0 | 0 |
| g-Valerolactone | 0.05 | 0.3356 | 0 | 0 |
| Methanol | 0.05 | 0.3356 | 0 | 0 |

**Note:** * Reaction time was 0.5 h. STY refers to the site time yield calculated as the moles of product per mol of catalyst per unit of time.

**2.4.4. Catalyst stability in aqueous environments.** In general, when conducting catalytic reactions in aqueous media the polar solvent may affect the catalyst performance by several possible phenomena that include; the irreversible adsorption (poisoning), the dissolution of the active site into the bulk water phase (lixiviation),[9,10] the destruction of the crystalline structure,[22–24] and combinations thereof. In order to evaluate the stability of $WO_3$ commercial catalyst we evaluated the extent of lixiviation into the liquid solution under reactive conditions at pH of 6.4. As shown in **Fig. S4** the fraction of $WO_3$ that lixiviated into the solution reached a plateau after 3 h of reaction (~12 wt. %). The dissolution of tungsten oxide species at pH 6.4 is favored due to high solubility of tungsten oxide in basic medium.

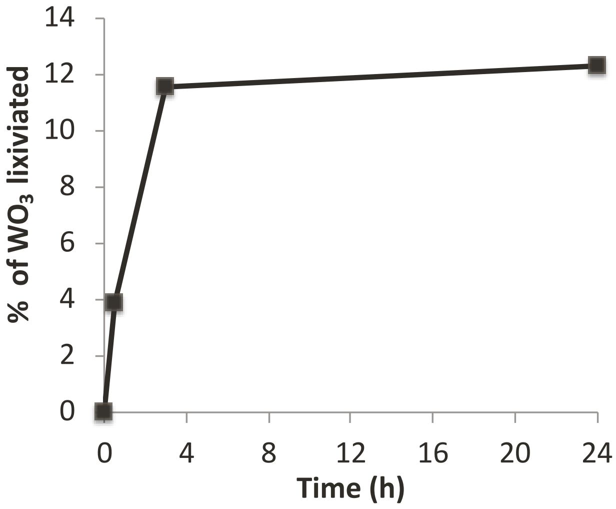


**Figure S4:** Extent leaching of $WO_3$ Commercial catalyst as a function of time at pH 6.4 Reaction conditions: Concentration of Crotonic acid 0.25 M; Reaction temperature 65 ºC; Reaction time 0.5 hours; Volume of Oxidant 230 µL of H2O2 at 10 % (v/v); and Amount of catalyst 50 mg.

In view of these results, we performed a two steps experiment to determine the extent of the reaction that is accomplished by the dissolved tungsten oxide during epoxidation at pH 2.9 and 6.4 (see **Table 2**). In the first step, the reaction was performed for ten minutes in the presence of $WO_3$ catalyst (Exp.1) and the catalyst solids were separated from solution and the concentration of crotonic acid was measured. In the second step, the liquid fraction was placed back in the reaction system and the reaction was allowed to proceed for an additional twenty minutes (Exp. 2). The final conversion attained after the two steps was calculated and compared with an experiment of thirty minutes in which the catalyst was continuously in contact with the reaction mixture (Exp. 3). The extent of homogeneous catalysis was calculated as shown in **Equation 3**.

$$\%Homogeneous\ Rxn. = \frac{(X_{exp.2} - X_{exp.1})}{X_{exp.3}} * 100 \qquad \textbf{Eq. 3}$$

The results in **Table S6** indicate that regardless of the pH 2.9 and 6.4 and catalyst employed the activity continued after the solid catalyst was removed, which indicates that the fraction of dissolved $WO_3$ is catalytically active. However, the extent of the homogeneous reaction varied depending on the nature of the catalyst and the pH. For instance, at low pH (2.9) the extent of homogeneous reaction was 22.5 and 10.0 % for the $WO_3$ catalyst synthesized by combustion and the $WO_3$-SBA-15, respectively. At pH 6.4 these values significantly increased reaching values of 60.0 and 22.6% for $WO_3$ combustion and $WO_3$-SBA-15 catalysts, correspondingly, which could be attributed to higher concentration of dissolved $WO_3$ at high pH. Notably, when the reaction was performed using the $WO_3$-SBA-15 the extent of homogeneous catalyzed reaction was significantly smaller than that of the $WO_3$ prepared by combustion.

**Table S6.** Extent of conversion after catalyst removal from the reaction mixture as a function of the pH of the solution. The epoxidation of crotonic acid was performed at 65 °C and atmospheric pressure using 230 µL of $H_2O_2$ at 10 vol. % on 0.05 g of $WO_3$ combustion and $WO_3$-SBA-15 catalysts (no base was added in these experiments).

| Catalyst | pH | Conversion (%) | | | Extent of homogeneous Reaction (%) |
|---|---|---|---|---|---|
| | | Exp. 1 | Exp. 2 | Exp. 3 | |
| $WO_3$ Combustion | 2.9 | 28 | 40 | 52 | 22.5 |
| | 6.4 | 25 | 78 | 89 | 60.0 |
| $WO_3$-SBA-15 | 2.9 | 18 | 21 | 32 | 10.0 |
| | 6.4 | 19 | 30 | 48 | 22.6 |

### 2.5. Hydrogenation of epoxy acid

**2.5.1. Two step hydrogenation using Pd/C followed by $NaBH_4$ treatment.** To perform the product identification of the 3-hydroxybutanoic acid, we decided to employ a two-step hydrogenation step of the epoxide product mixture using hydrogenation on Pd/C (Sigma-Aldrich) in continuous hydrogen bubbling at room temperature for 24 h followed by a final hydrogenation step in aqueous solution of $NaBH_4$ (50 mg). In this case, the reaction of epoxidation was performed for 24 hr at pH of 6.4, which led to the formation of significant quantities of other products besides the epoxy acid and the di-hydroxy acid (**Fig. S5**). The $^1$H-NMR (a) and $^{13}$C-NMR (b) spectra confirmed the presence of the 3-methyloxirane-2-carboxylic to 3-hydroxybutyric acid, with the concomitant formation of butanoic acid from crotonic acid. The presence of additional products led to appearance of additional NMR peaks. As it is shown in **Fig. S6a**, after the 24 h epoxidation reaction crotonic acid (29.9 min) was successfully converted to 3-methyloxirane-2-carboxylic acid (17.2 min) and 2,3-dihydroxy-butanoic acid (8.4 min). After the two steps hydrogenation process, we successfully formed 3-hydroxy-butanoic acid (13.5 min) in combination with saturated $\alpha,\beta$-unsaturated acid to 2,3-dihydroxybutanoic acid. The combination of HPLC-RI and NMR characterization helped us to undoubtedly identify the successful conversion of the 3-methyloxirane-2-carboxylic acid to 3-hydroxybutyric acid and 2,3-dihydroxybutanoic acid.

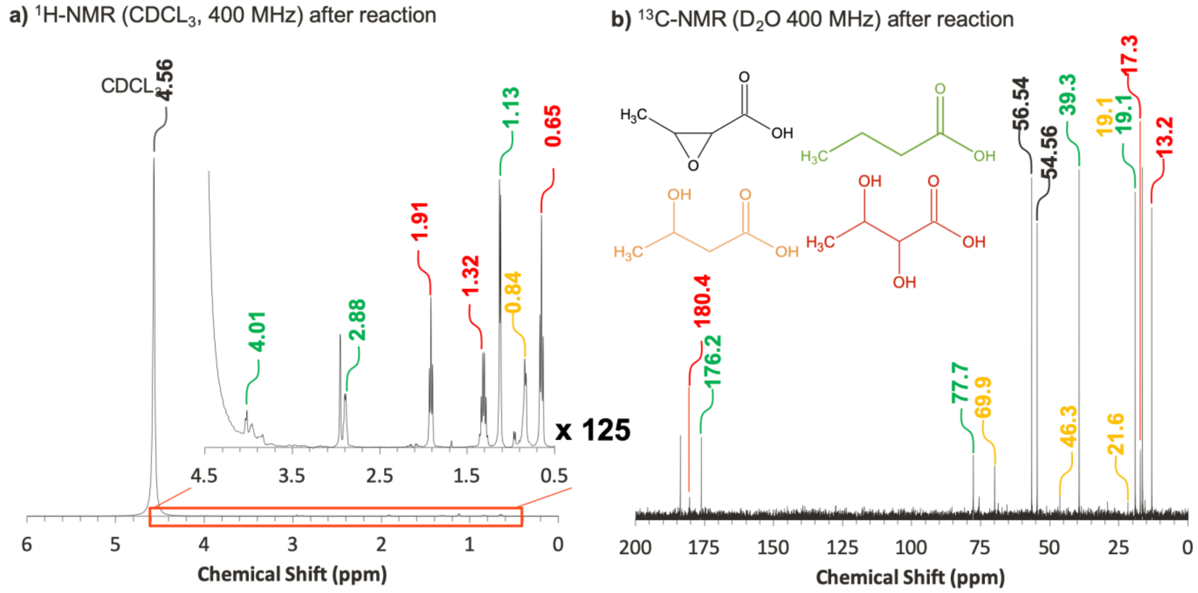


**Figure S5.** $^1$H-NMR (a) and $^{13}$C-NMR (b) spectra of the epoxidation reaction (24 h) products after batch hydrogenation of the epoxy acid product mixture obtained after 24 h of reaction at room temperature using $H_2$ and Pd/C followed by treatment in an aqueous solution of $NaBH_4$ (50 mg).

a)

b)

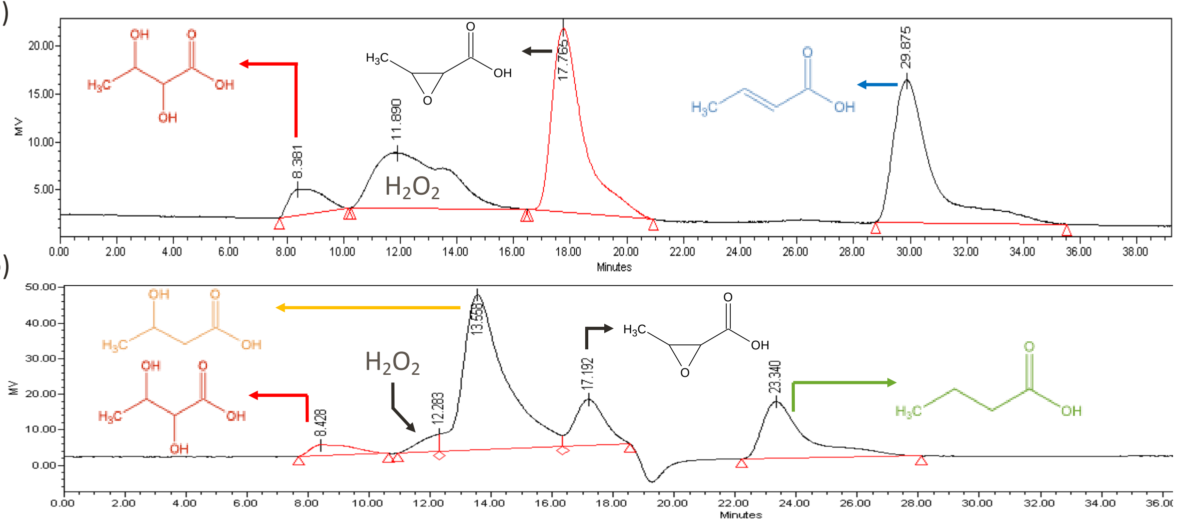


**Figure S6.** HPLC-RI analysis of the reaction mixture obtained after 24 h of epoxidation of crotonic acid on 50 mg of $WO_3$ synthesized by the combustion method (a). The epoxidation was performed at an initial pH of 6.4, 65 C, and 230 µL of $H_2O_2$ at 10 vol. %. The hydrogenation of the epoxy acid containing mixture was performed on 50 mg of Pd/C (Sigma-Aldrich) for 24 h at room temperature and 1 bar of $H_2$ in continuous bubbling through the reaction mixture followed by a second hydrogenation step in an aqueous solution (15 mL) of $NaBH_4$ (50 mg) (b).

**2.5.2. Epoxide extraction followed by hydrogenation by $NaBH_4$.** To further demonstrate the correct identification of the 3-hydroxybutanoic acid we performed an experiment consisting in the extraction of the epoxy acid followed by a single hydrogenation step using $NaBH_4$ in aqueous environment. The process started with the crotonic acid epoxidation using 50 mg of $WO_3$ synthesized by the combustion method for 24 h at an initial pH of 6.4, 65 °C, and 230 µL of $H_2O_2$ at 10 vol. %. The oxidized products were neutralized with dilute sulfuric acid and supersaturated with ammonium sulfate salt. The free epoxy acid product was extracted with diethyl ether and the solvent was evaporated to dryness to obtain crude solid product and analysed by HPLC to confirm the purity of the product (**Fig. S7a**). The chromatogram indicated the presence of only two species in the solution that corresponded to the 3-methyloxirane-2-carboxylic acid (17.5 min) and the crotonic acid (29.8 min).

The final solid product was diluted with water and hydrogenated with sodium borohydride agent at room temperature (**Fig. S7b**). The HPLC chromatogram of the hydrogenation product indicated the formation of 3-hydroxy-butanoic acid (13.5 min) followed by unreacted 3-methyloxirane-2-carboxylic acid (17.5 min) and the crotonic acid (29.8 min). The conversion of the hydrogenation step was 40 % with 100 % selectivity towards the saturation of the epoxy ring at the alpha position, which resulted in the β-hydroxy acid.

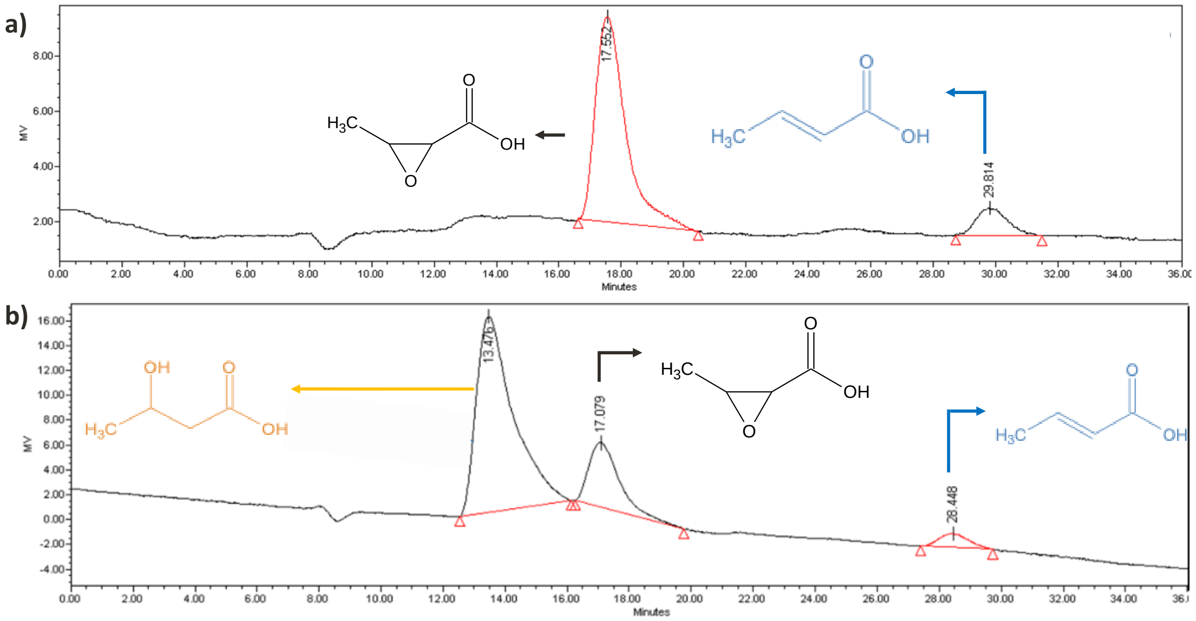


**Figure S7.** HPLC-RI analysis of the reaction mixture obtained after 24 h of epoxidation of crotonic acid on 50 mg of $WO_3$ synthesized by the combustion method followed by extraction **(a)**. The hydrogenation of the extracted epoxy acid was performed on 50 mg of $NaBH_4$ (50 mg) for 2 h **(b)**.

**2.5.3. Hydrogenation on 5 wt. % Ru/C.** Analysis of the epoxide containing mixture before the hydrogenation on 5 wt. % Ru/C (Sigma-Aldrich) indicated the presence of unreacted crotonic acid (29.9 min) and hydrogen peroxide (11.9 min) in combination with the epoxidation product (3-methyloxirane-2-carboxylic acid) at 17.5 min and 2,3-dihydroxybutanoic acid (8.4 min) resulting from hydration of the epoxide. After 2h or reaction on the 5 wt. % Ru/C (Sigma-Aldrich) we observed the formation of 3-hydroxy-butanoic acid (13.5 min), and butanoic acid (23.4 min) in combination with the unreacted 2,3-dihydroxy-butanoic acid (8.4 min) (**Fig. S8**).

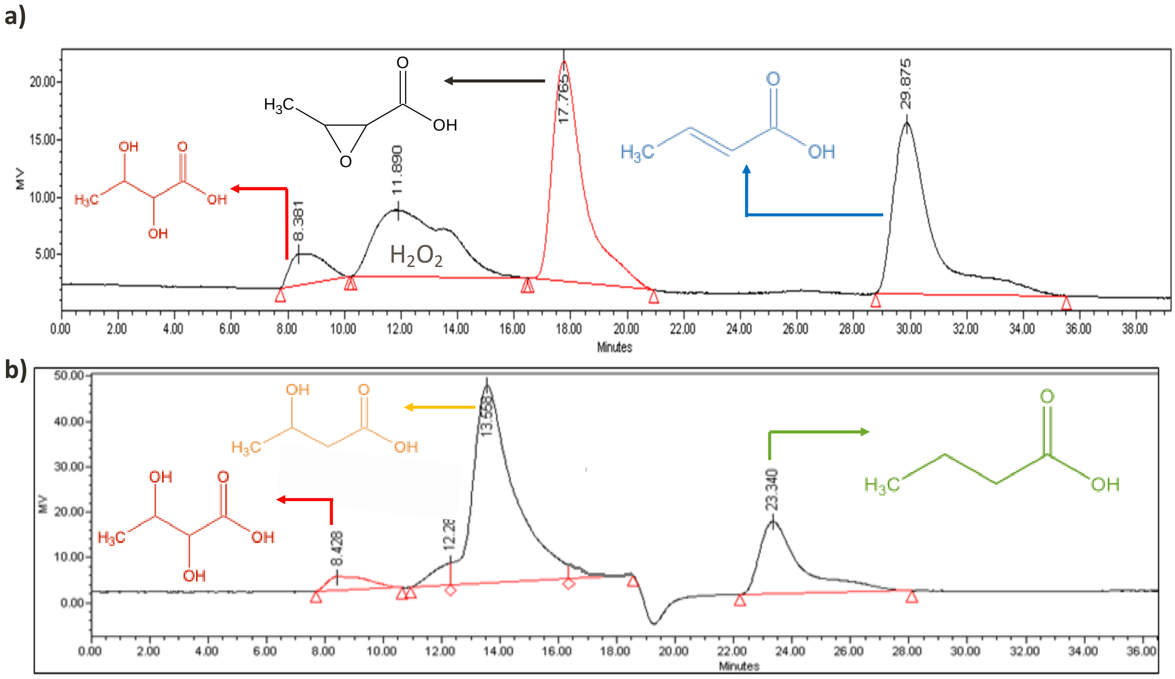


**Figure S8.** HPLC-RI analysis of the reaction mixture obtained after 2 h of epoxidation of crotonic acid on 50 mg of $WO_3$ synthesized by the combustion method **(a)** followed by the hydrogenation of epoxy acid containing mixture using 50 mg of 5 wt. % Ru/C (Sigma-Aldrich) at 180 ºC, 34 bar of $H_2$ and 800 rpm of agitation **(b)**.

## 3. Product characterization by $^1$H- and $^{13}$C- NMR.

**Crotonic acid.** $^1$H NMR (400 MHz, $D_2O$): δ 1.85 (d, $J_{HH}$ = 6.7 Hz, 3H), δ 5.87 (d, $J_{HH}$ =15.6 Hz, 1H), δ 7.02 (sex, $J_{HH}$ = 15.7 Hz, 1H). $^{13}$C NMR (400 MHz, $D_2O$): δ 17.3, δ 121.2, δ 148.3, δ 170.

**3-methyloxirane-2-carboxylic acid (Epoxidation product).** $^1$H NMR (400 MHz, $CDCl_3$): δ 1.31 (d, $J_{HH}$ = 5.4 Hz, 3H), δ 3.1 (d, $J_{HH}$ = 8.4 Hz, 1H), δ 3.15 (dq, $J_{HH}$ =8.1 Hz, $J_{HH}$ = 5.4 1H). $^{13}$C NMR (400 MHz, $D_2O$): δ 16.2, δ 54.4, δ 56.43, δ 175.6.

**3-hydroxybutanoic acid (β-Hydroxy butanonic acid).** $^1$H NMR (400 MHz, $CDCl_3$): δ 1.129 (d, $J_{HH}$ = 5.4 Hz, 3H), δ 2.88 (d, $J_{HH}$ = 5.34 Hz, 2H), δ 4.01 (sex, $J_{HH}$ = 6.1 Hz, 1H). $^{13}$C NMR (400 MHz, $D_2O$): δ 19.1, δ 39.34, δ 77.7, δ 176.2.

**Butanoic acid (Direct hydrogenation of crotonic acid).** $^1$H NMR (400 MHz, $CDCl_3$): δ 0.65 (d, $J_{HH}$ = 7.9 Hz, 3H), δ 1.32 (sex, $J_{HH}$ = 7.2 Hz, 2H), δ 1.91 (t, $J_{HH}$ = 7.1 Hz, 2H). $^{13}$C NMR (400 MHz, $D_2O$): δ 13.2, δ 17.3, δ 180.4.

**2,3-dihydroxybutanoic acid (Dihydroxy product).** $^1$H NMR (400 MHz, $CDCl_3$): δ 0.84 (d, $J_{HH}$ = 5.83 Hz, 3H), δ 4.1 (quin, $J_{HH}$ = 6.17 Hz, 1H). $^{13}$C NMR (400 MHz, $D_2O$): δ 19.1, δ 69.9, δ 77.69, δ 178.2.

## 4. Preliminary techno-economic analysis:

### 4.1. Conceptual process design.

In **Figure S9** it is presented the process flow diagram for the conversion of primary alcohols, such as bioethanol, into high-purity building blocks (unsaturated acids, mono-hydroxy acids, and di-hydroxy acids). Initially, a stream (10) containing primary alcohols such as bio-ethanol is fed directly to the reactor (2), in which dehydrogenation or oxidation reaction takes place to convert the primary alcohol into an aldehyde. Alternatively, stream (10) can be pretreated in the guard bed (1) to produce an impurities-free stream (12) before entering oxidative dehydrogenation reactor (2). The product of this unit could be fractionated into acetaldehyde and ethanol in the distillation unit 2(a) to recycle the unreacted feed. Alternatively, the entire product stream could be sent to the aldol-condensation reaction unit (3), in which the catalyst [13] is employed to selectively convert the aldehyde-containing stream (13) into longer unsaturated aldehydes via aldol-condensation. Reactor (3) can accept an external source of aldehyde:primary alcohol mixture produced elsewhere using stream (14). The stream containing unsaturated aldehydes (15) is sent to a distillation unit (4) that separates unconverted aldehyde and primary alcohol from the unsaturated aldehydes. The unconverted aldehyde and primary alcohol stream (16) are recycled back to reactor (2). The unsaturated aldehydes stream (17) is fed to reactor (5). In reactor (5) the catalyst [14] oxidizes the unsaturated acid stream (17) into unsaturated acids. The unsaturated acids stream (18) can be sent to the separation unit (6) to produce a high-purity unsaturated acids stream (19), and stream (20) of unreacted unsaturated aldehyde is recycled back to reactor (5).

Alternatively, the stream (21) containing unsaturated acids can be employed to produced mono- or di-hydroxy acids depending on the reaction conditions utilized. If the desired product is the mono-hydroxy acid then stream (21) is processed in reactor (7) using catalyst [15] in the presence of a pH modifying agent (i.e. pH near 7) continuously fed by stream (22), which facilitates selective epoxidation of the unsaturated acid to epoxy acids. The stream (23) containing epoxy acid is sent to reactor (8) where a hydrogenation catalyst is employed to produce mono-hydroxyacids (i.e. β-hydroxy acids). The outlet of reactor (8) containing mono-hydroxyacids (24) is fed to a separation unit (9) that produces high-purity mono-hydroxy acids in stream (25) and recycling products in stream (26), which are fed back to reactor (8). Alternatively, if the desired product is di-hydroxy acid then the stream (21) containing unsaturated acid is fed to reactor (7) using catalyst [15] in the absence of a pH modifying agent (i.e. low pH), which maximizes the production of 2,3-dihydroxy acids. The di-hydroxy acids produced in reactor (7) can be either saturated or unsaturated di-hydroxy acids depending on the reaction conditions employed. If the product from reactor (7) contains saturated di-hydroxy acids, then reactor (8) can be bypassed and stream (27) containing saturated dihydroxy acids can be sent directly to separation unit (9) to produce high purity di-hydroxy acids in stream (25). In the case of unsaturated dihydroxy acids the stream (23) containing unsaturated dihydroxy acids can be fed to the hydrogenation reactor (8) to produce the saturated di-hydroxy acid. Finally, the resulting stream (24) comprising of saturated di-hydroxy acids is fed to the separation unit (9) to produce stream (25) containing saturated di-hydroxy acids and stream (26) containing unreacted unsaturated dihydroxy acids, which are recycled back to reactor sent (8).

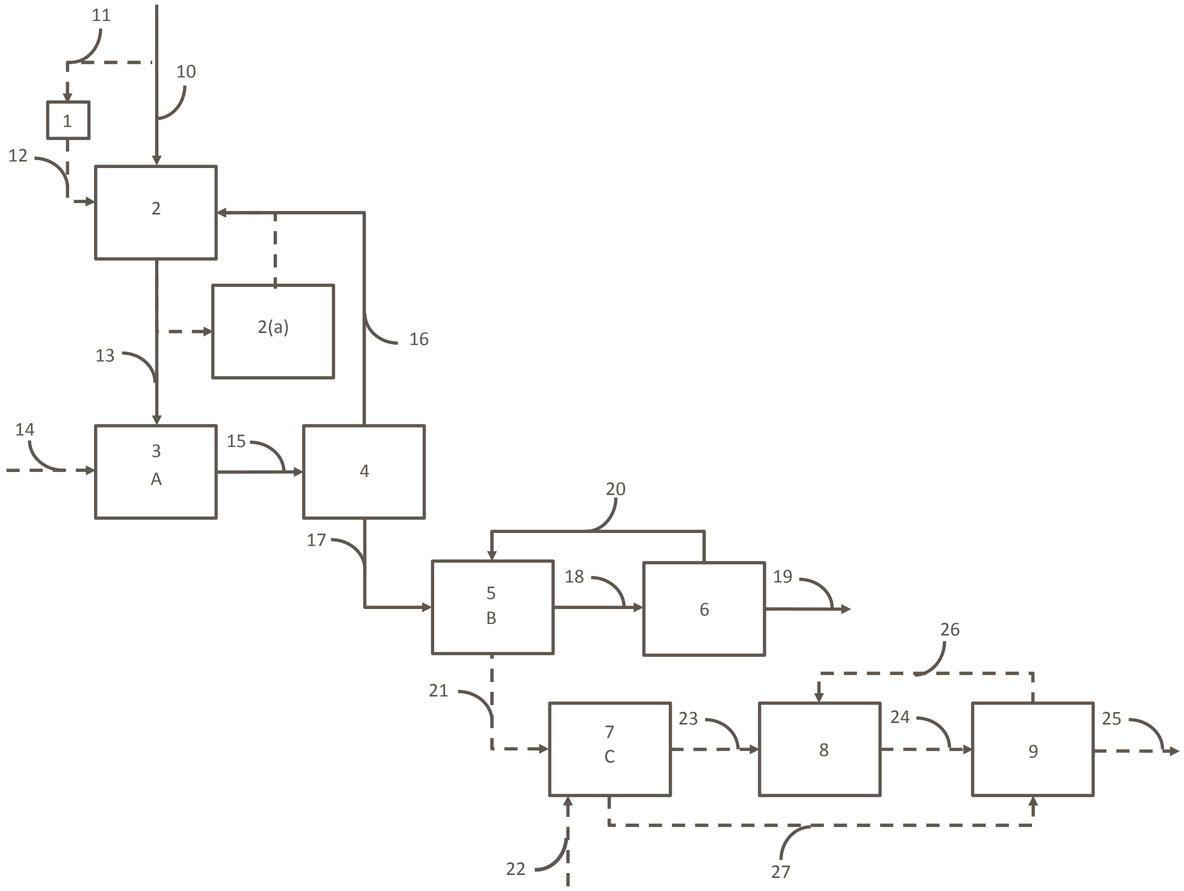


**Figure S9:** Process flow diagram for the conversion of renewable ethanol into high-purity building blocks (unsaturated acids, mono-hydroxy acids, and di-hydroxy acids).

### 4.2. Distillation resistance

To estimate the cost of the crotonic acid, 3-hydroxybutanoic acid, and 3-methyloxirane-2-carboxylic acid we employed the approach reported by J. P. Lange in which the concept of "*distillation resistance"* was employed to assess the potential costs of purification at the very early stage of the research.[25] While this methodology does not replace a full-technoeconomic assessment it allow us to pin-point the main issues that can should be address in the process to improve its economic feasibility.

In this methodology, the first step is to calculate the so-called distillation resistance (Ω) using **Equation 4**. For this we ordered the molecules that were present in the mixture by increasing normal boiling point. The resulted values Ω of the feed are then employed to obtain the Ω of the product using the mass fraction of the products (**Equation 5**). Assuming a plant capacity of 100x10$^3$ ton per year, which is reasonable for a biorefinery process,[25–27] we employed an empirical relationship that correlates the plant capacity and the distillation resistance for the product (**Eq. 6**).

$$\Omega_{Feed}(°\mathrm{C}^{-1}) = 100 \times \sum_{i=1}^{n}\left(\frac{f_i(W/W)}{\Delta T_i(°\mathrm{C})}\right)$$ **Eq. 4**

$$\Omega_{Prod}(°\mathrm{C}^{-1}) = \frac{\Omega_{Feed}(°\mathrm{C}^{-1})}{f_{product}}$$ **Eq. 5**

$$D_C\left(\frac{\$}{Ton_{product\ 2003}}\right) = \frac{60\times[\Omega_{Prod}(°\mathrm{C}^{-1})]^{0.65}}{\left[Capacity\left(\frac{kTon}{Year}\right)\right]^{0.35}} + 3 \times \Omega_{Prod}(°\mathrm{C}^{-1})$$ **Eq. 6**

The results of these calculations are presented in the **Table S7-S10**.

**Table S7:** Distillation resistance of the feed and product for the distillation **2a** in the process scheme shown in **Figure S9**. It was assumed that any incompressible gas is separated perfectly form the mixture in a flash tank before entering the distillation column **2(a)**.

| **Distillation #1 (unit 2(a) –Figure S9)** | | | | | |
|---|---|---|---|---|---|
| **Molecule** | **Concentration (wt. %)** | **BP (°C)** | **ΔDT (°C)** | **Ω Feed** | **Ω Product** |
| Acetaldehyde | 15.59 | 20.20 | 58.17 | 0.27 | 1.72 |
| Ethanol | 78.04 | 78.37 | 21.63 | 3.61 | 23.15 |
| Water | 6.37 | 100.00 | | | |
| | | | **Total** | **3.88** | **24.86** |
| **Fraction of C4 aldehydes (%)** | | | | | **16** |
| **Distillation Cost_1 ($/ton of Product)** | | | | | **171** |

**Table S8:** Distillation resistance of the feed and product for the stream **16** entering the distillation column **4** in the process scheme shown in **Figure S9**. It was assumed that these molecules are thermally stable at the conditions employed for the separation.

| **Distillation #2 (unit 4 –Figure S9)** | | | | | |
|---|---|---|---|---|---|
| **Molecule** | **Concentration (wt. %)** | **BP (°C)** | **ΔDT (°C)** | **Ω Feed** | **Ω Product** |
| Acetaldehyde | 4.44 | 20.20 | 54.60 | 0.08 | 0.90 |
| Butanal | 0.62 | 74.80 | 2.30 | 0.27 | 2.98 |
| Ethylacetate | 1.04 | 77.10 | 1.27 | 0.82 | 9.03 |
| Ethanol (Solvent) | 82.26 | 78.37 | 23.63 | 3.48 | 38.50 |
| 1,1-Diethoxyethane | 0.20 | 102.00 | 2.00 | 0.10 | 1.13 |
| Crotonaldehyde | 0.92 | 104.00 | 13.70 | 0.07 | 0.74 |
| Butanol | 0.82 | 117.70 | 3.50 | 0.23 | 2.60 |
| Crotyl alcohol | 8.12 | 121.20 | 52.80 | 0.15 | 1.70 |
| Mixed C6 | 0.26 | 174.00 | 41.00 | 0.01 | 0.07 |
| Mixed C8 | 1.32 | 215.00 | | | |
| | | | **Total** | 5.20 | 54.25 |
| **Fraction of C4 aldehydes (%)** | | | | | **9** |
| **Distillation Cost_2 ($/ton of Product)** | | | | | **340** |

**Table S9:** Distillation resistance of the feed and product for the stream **20** entering the distillation column **6** in the process scheme shown in **Figure S9**. It was assumed that these molecules are thermally stable at the conditions employed for the separation.

| **Distillation #3 (unit 6 –Figure S9)** | | | | | |
|---|---|---|---|---|---|
| **Molecule** | **Concentration (wt. %)** | **BP (°C)** | **ΔDT (°C)** | **Ω Feed** | **Ω Product** |
| Croton aldehyde | 1.03 | 104.00 | 14.10 | 0.07 | 0.57 |
| Acetic acid | 86.12 | 118.10 | 66.60 | 1.29 | 10.07 |
| Crotonic Acid | 12.85 | 184.70 | 0.00 | 0.00 | 0.00 |
| | | | **Total** | 1.37 | 10.64 |
| **Fraction of Crotonic Acid (%)** | | | | | **13** |
| **Distillation Cost_3 ($/ton of Product)** | | | | | **88** |

**Table S10:** Distillation resistance of the feed and product for the stream **26** entering the distillation column **9** in the process scheme shown in **Figure S9**. It was assumed that these molecules are thermally stable at the conditions employed for the separation.

| **unit 9** | | | | | |
|---|---|---|---|---|---|
| **Molecule** | **Concentration (wt. %)** | **BP (°C)** | **ΔDT (°C)** | **Ω Feed** | **Ω Product** |
| Water | 96.36 | 100.00 | 64.00 | 1.51 | 76.12 |
| Butanoic acid | 1.23 | 164.00 | 105.20 | 0.01 | 0.00 |
| 3-hydroxy-butyric acid | 1.98 | 269.20 | 89.60 | 0.02 | 0.00 |
| 2,3-dihydroxybutanoic acid | 0.43 | 358.80 | | | |
| | | | **Total** | 1.54 | 76.12 |
| **Fraction of 3-hydroxybutanoic acid (%)** | | | | | **2** |
| **Distillation Cost_4 ($/ton of Product)** | | | | | **428** |

Notably, from this preliminary cost analysis we can pinpoint that the main contributors to the final cost of purifying crotonic acid and 3-hydroxybutanoic acid are the separations required after the aldol-condensation to purify the C4- unsaturated aldehydes (340 $/ton) and the final distillation of the β-hydroxy acid after the hydrogenation (428 $/ton). The high costs of distillation are primarily due to the low concentration of these molecules in the product streams. This results in large distillation resistances as it can be seen by the large vales of Ω product (>50).

By assuming a cost of ethanol of one dollar per liter we estimated a cost of 3-hydroxybutanoic acid of 3.1 $/Kg (2.8 €/Kg conversion factor of 1.1 €/$), while the cost of crotonic acid is estimated at 2.8 $/Kg (2.6 €/Kg conversion factor of 1.1 €/$). As it is shown in **Figure S10**, the cost of the intermediate epoxide (3-methyloxirane-2-carboxylic acid) is higher than that of the α,β-hydroxy acid. The main reason for this is the higher weight yields of the latter. While it is true that these costs include the contributions of the feed and separation costs (energy and capex amortization), other costs associated with the amortization of the reactors, catalysts, and the energy associated with the conversion are not included. Nevertheless, all the conversion steps involved in our process are exothermic (oxidative dehydrogenation, aldol-condensation, oxidation, epoxidation, hydrogenation), so we do not expect significant energy costs associated with the conversion steps.

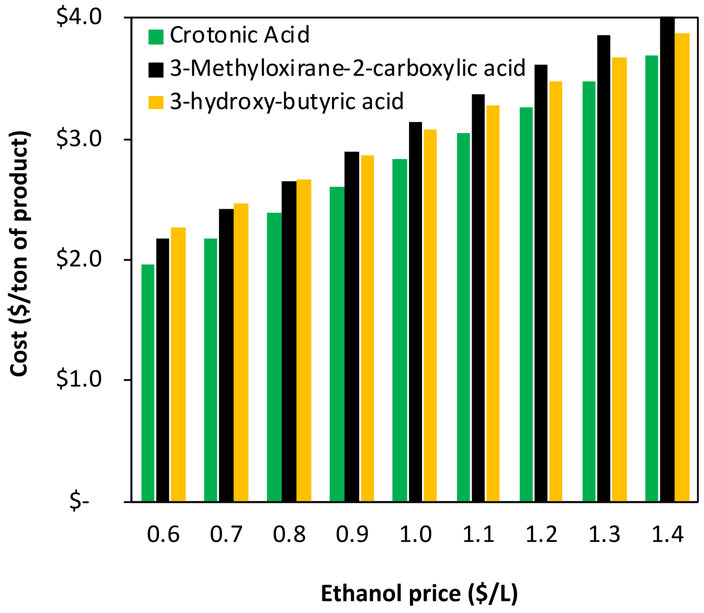


**Figure S10:** Sensitivity analysis of the cost of crotonic acid, 3-methyloxirane-2-carboxylic acid, and 3-hydroxy-butyric acid as a function of ethanol price.

## 5. References


[1] E. Flahaut, C. Laurent, A. Peigney, *Carbon N. Y.* **2005**, *43*, 375–383.
[2] Q. Tang, Q. Zhang, P. Wang, Y. Wang, H. Wan, *Chem. Mater.* **2004**, *16*, 1967–1976.
[3] L. Zhang, T. N. Pham, J. Faria, D. Santhanaraj, T. Sooknoi, Q. Tan, Z. Zhao, D. E. Resasco, *ChemSusChem* **2016**, *9*, DOI 10.1002/cssc.201501518.
[4] H. Ji, T. Mizugaki, K. Ebitani, K. Kaneda, *Tetrahedron Lett.* **2002**, *43*, 7179–7183.
[5] T. N. Pham, L. Zhang, D. Shi, M. R. Komarneni, M. P. Ruiz, D. E. Resasco, J. Faria, *ChemCatChem* **2016**, *8*, 1–11.
[6] S. Fujita, N. Iwasa, H. Tani, W. Nomura, M. Arai, N. Takezawa, *React. Kinet. Catal.* **2001**, *73*, 367–372.
[7] S. Tayrabekova, P. Mäki-Arvela, M. Peurla, P. Paturi, K. Eränen, G. E. Ergazieva, A. Aho, D. Y. Murzin, K. Dossumov, *Comptes Rendus Chim.* **2018**, *21*, 194–209.
[8] T. N. Pham, L. Zhang, D. Shi, M. R. Komarneni, M. P. Ruiz, D. E. Resasco, J. Faria, *ChemCatChem* **2016**, *8*, DOI 10.1002/cctc.201600953.
[9] F. Héroguel, B. Rozmysłowicz, J. S. Luterbacher, *Chim. Int. J. Chem.* **2015**, *69*, 582–591.
[10] H. Xiong, H. N. Pham, A. K. Datye, *Green Chem.* **2014**, *16*, 4627–4643.
[11] A. L. Jongerius, J. R. Copeland, G. S. Foo, J. P. Hofmann, P. C. A. Bruijnincx, C. Sievers, B. M. Weckhuysen, *ACS Catal.* **2013**, *3*, 464–473.
[12] N. Aranda-Pérez, M. P. Ruiz, J. Echave, J. Faria, *Appl. Catal. A Gen.* **2017**, *531*, DOI 10.1016/j.apcata.2016.10.025.
[13] E. Briot, J. Y. Piquemal, M. Vennat, J. M. Brégeault, G. Chottard, J. M. Manoli, *J. Mater. Chem.* **2000**, *10*, 953–958.
[14] B. Weng, J. Wu, N. Zhang, Y. J. Xu, *Langmuir* **2014**, *30*, 5574–5584.
[15] J. Zhang, L. Zhang, X. Shen, P. Xu, J. Liu, *CrystEngComm* **2016**, *18*, 3856–3865.
[16] F. Wang, Y. Wang, X. Zhan, M. Safdar, J. Gong, J. He, *CrystEngComm* **2014**, *16*, 1389–1394.
[17] A. Srinivasan, M. Miyauchi, *J. Phys. Chem. C* **2012**, *116*, 15421–15426.
[18] G. B. Payne, P. H. Williams, *J. Org. Chem.* **1959**, *24*, 54–55.
[19] R. Webber, *J. Catal.* **1995**, *151*, 470–474.
[20] E. Iglesia, D. G. Barton, S. L. Soled, S. Miseo, J. E. Baumgartner, W. E. Gates, G. A. Fuentes, G. D. Meitzner, *Stud. Surf. Sci. Catal.* **1996**, *101*, 533–542.
[21] C. Hammond, J. Straus, M. Righettoni, S. E. Pratsinis, I. Hermans, *ACS Catal.* **2013**, *3*, 321–327.
[22] P. A. Zapata, J. Faria, M. P. Ruiz, R. E. Jentoft, D. E. Resasco, *J. Am. Chem. Soc.* **2012**, *134*, 8570–8578.
[23] D. Shi, J. Faria, T. N. Pham, D. E. Resasco, *ACS Catal.* **2014**, *4*, 1944–1952.
[24] N. Aranda-Pérez, M. Pilar Ruiz, J. Echave, J. Faria, *Appl. Catal. A Gen.* **2017**, *531*, 106–118.
[25] J. P. Lange, *ChemSusChem* **2017**, *10*, 245–252.
[26] J. P. Lange, *Angew. Chemie - Int. Ed.* **2015**, *54*, 13187–13197.
[27] J. P. Lange, *Catal. Sci. Technol.* **2016**, *6*, 4759–4767.
